# The Interprocessual-Self Theory in Support of Human Neuroscience Studies

**DOI:** 10.3389/fpsyg.2021.686928

**Published:** 2022-01-28

**Authors:** Elkin O. Luis, Kleio Akrivou, Elena Bermejo-Martins, Germán Scalzo, José Víctor Orón

**Affiliations:** ^1^Psychological Processes in Education and Health Group, School of Education and Psychology, University of Navarra, Pamplona, Spain; ^2^Navarra Institute for Health Research, Pamplona, Spain; ^3^Henley Business School, University of Reading, Reading, United Kingdom; ^4^Department of Community Nursing and Midwifery, School of Nursing, University of Navarra, Pamplona, Spain; ^5^School of Business, Universidad Panamericana, Mexico City, Mexico; ^6^Fundación UpToYou Educación, Zaragoza, Spain

**Keywords:** interprocessual-self theory, interpersonal relationships, personal growth, neuroscience, self-knowledge, integration, agency

## Abstract

Rather than occurring abstractly (autonomously), ethical growth occurs in interpersonal relationships (IRs). It requires optimally functioning cognitive processes [attention, working memory (WM), episodic/autobiographical memory (AM), inhibition, flexibility, among others], emotional processes (physical contact, motivation, and empathy), processes surrounding ethical, intimacy, and identity issues, and other psychological processes (self-knowledge, integration, and the capacity for agency). Without intending to be reductionist, we believe that these aspects are essential for optimally engaging in IRs and for the personal constitution. While they are all integrated into our daily life, in research and academic work, it is hard to see how they are integrated. Thus, we need better theoretical frameworks for studying them. That study and integration thereof are undertaken differently depending on different views of what it means to live as a human being. We rely on neuroscientific data to support the chosen theory to offer knowledge to understand human beings and interpersonal relational growth. We should of course note that to describe what makes up the uniqueness of being, acting, and growing as a human person involves something much more profound which requires too, a methodology that opens the way for a theory of the person that responds to the concerns of philosophy and philosophical anthropology from many disciplines and methods (Orón Semper, 2015; Polo, 2015), but this is outside the scope of this study. With these in mind, this article aims to introduce a new explanatory framework, called the Interprocessual-self (IPS), for the neuroscientific findings that allow for a holistic consideration of the previously mentioned processes. Contributing to the knowledge of personal growth and avoiding a reductionist view, we first offer a general description of the research that supports the interrelation between personal virtue in IRs and relevant cognitive, emotional, and ethic-moral processes. This reveals how relationships allow people to relate ethically and grow as persons. We include conceptualizations and descriptions of their neural bases. Secondly, with the IPS model, we explore neuroscientific findings regarding self-knowledge, integration, and agency, all psychological processes that stimulate inner exploration of the self concerning the other. We find that these fundamental conditions can be understood from IPS theory. Finally, we explore situations that involve the integration of two levels, namely the interpersonal one and the social contexts of relationships.

## Introduction

Developed as a cross-disciplinary concept (Akrivou and Orón, 2016; Akrivou et al., 2018) inspired by Polo’s anthropology (Polo, 1971, 1999), Interprocessual-self (IPS) is a novel theory of the self and action. According to it, Western civilization has been profoundly influenced by three fundamental roots (radicals), namely the Greek/classical radical or “radical of nature” (represented by Aristotle, it stresses human nature in its biological and psychological aspects, which can be perfected by virtuous growth); the modern radical or the “radical of the subject” (emphasizing production and novelty, and manifesting cognition that achieves mastery through production); and the Christian radical or the “radical of the person” (emphasizing the human person through her singularity and relational intimacy, as well as the notion of co-existence as constitutive of the person) (Polo, 1999). IPS conceptualizes an integration of these three radicals from within the radical of the person (Akrivou and Orón, 2016); this is based on the superiority of the radical of the person, which gives meaning to and orients the other two radicals by emphasizing human singularity via intimate relations (freedom for) and our immense possibilities for personal and interpersonal growth. This explains why the radicals are hierarchically linked, while also constituting three distinct ways of capturing important parts of human beings (González, 2018) that simultaneously involve both tensions and compatibilities.

This theory comes from moral psychology and centers on human development theory. It is based on the unity between knowledge and action (virtue) rooted in personalist anthropology; as such, it elevates the person’s deep disposition toward growth through acts of intimacy and humanity, which enable uniqueness (novelty) in personal and interpersonal relations that are constitutive of ethical progress and innovation. IPS transcends modernist human development proposals— developed in the last 50 years in educational, psychological, and adult development research— that mainly acknowledge the ‘modern radical’ (Orón Semper et al., 2019). Modern philosophy and psychology’s focus on the modern radical to the exclusion of the radical of nature and of the person loses sight of human beings and serves as a very limited conceptual background for achieving unified knowledge of neuroscientific data (Akrivou and Orón, 2016). This proposal, with IPS as its conceptual background, overcomes that limitation. IPS embraces IRs as the basis for personal and social growth whereby ethical being, knowledge, and action are at the heart of growth, integrating the three fundamental roots with a profound personalist viewpoint (Akrivou and Orón, 2016).

Interprocessual-self contains the following fundamental proposals:

First, IPS understands the person as coexisting *with*, where personal development occurs through the maturation and enrichment of personal intimacy accompanied by affective intensification. The IPS proposal draws on Polo’s (Polo and Corazón, 2005) transcendental anthropology (Akrivou et al., 2018) as a key theoretical source. It aims to integrate a transcendental personalist proposal with human action and growth on interrelational (IR), rather than autonomous, terms.

As such, it sees each person as in possession of absolute, inalienable value and of uniqueness that is expressed as intimacy, while significant personal relations enable said intimacy since personal growth is not an autonomous process, but rather happens through personal relations of significance. Thus, IPS theory is premised on coexistence: self-integrity is based on *personal coexistence with* and underlined by the notion of interpersonal self-donation (what Polo and Corazón, 2005 called “the personal radical”). However, both personal integration and personal action require the person to integrate this core (personal coexistence, personal relations) with two other spheres or roots related to who we are. This includes acting in our social dimension, which means acting as part of a wider community of flourishing (given that IPS is inclusive of virtue ethics, this implies attention to our common human nature and flourishing on the basis of character, virtue, and community [what Polo calls the “classical radical”]). It also includes acting while learning to incorporate human subjectivity and striving for novelty (called the “modern radical”). These three radicals also impact IPS’ focus on IR growth, implying an ethic whereby each person, as a complex singularity, acts toward growth that involves learning how to grow in terms of personal coexistence, or coexistence with others who are part of the three spheres described above. The idea of “personal coexistence” in IPS and personal integration of these three spheres requires and depends upon “personal giving and acceptance” of others and their donation. It also depends on the integration of cognitive, affective, ethical, and action-based components of the person in IR contexts, implying the different ways through which people affectively experience the self and donate the self in relationships.

The proposals that follow are based on the above and further expand key points surrounding IPS theory.

Second, according to IPS, the person grows in self-awareness precisely through the development of intimacy, thus increased intimacy in IRs makes a person who she is. Growth is both motivated by and signifies improvement of personal relationships via an epistemology of gift (Orón Semper, 2015, 2020). The relational dimension of this process is profound because personal ethical growth happens both ‘together with others’ and, importantly, ‘*for* the sake of others’ “in order to be able to love (others) better” (Fernández González, 2019, p. 27). Human action no longer rests on the development of particular cognitive faculties and instead rests on the conjunction of a multitude of unified cognitive-affective-practical-ethical faculties. Moreover, this unity is not an abstract object to be mastered by the actor (cognition as an object of agent), nor does the guiding subject have executive control over the actor (person/agent as an object of cognition). It is an integral part of a complicated reality called “person,” orienting action in a self-giving way. Its teleological end is the flourishing of all the parties who partake in a relationship and, at the same time, emphasizes process outcomes that attain overall growth. As such, growth is no longer subject to rules or objectives that must be reached “to be good at that ability,” but rather trusts *the person* as inherently relational.

Third, each person’s interiority is premised upon a transcendental anthropology (Arancibia, 2018; González, 2019). Fourth, self-integration of what we have learned about life (both positive and negative personal experiences), and the differentiation this entails (because each person integrates the multifaceted self differently), corresponds to two sides of the same phenomenon. Thus, integration, differentiation, identity, and growth are different ways of describing the same reality. Fifth, integration is a process of increasing self-understanding, and growth is based on one’s interiority, which requires relational intimacy (and which may involve some tension for persons with less experience). The fourth and fifth aspects of this theory orient personal action with a concern for development, which occurs when the person is what he is called to be in his own uniqueness and in terms of personal intimacy and relationships. This process is complicated and never linear. Thus, for IRs to work, they must enable shared growth among persons who wish to mutually support one another, growing as persons of virtue and engaging with intimacy and mutuality in an ongoing way.

These things are crucial not only for maintaining and promoting growth but also for supporting personal wellbeing. IRs are crucial for any personalist virtue ethics proposal (IPS, in this case). This approach is rooted in the person, who is the complex, basic atom of this theory. The personhood and development of the self-occur when the person aligns with her own uniqueness in personal relationships. As we will see, this is the basis for IPS’ emphasis on IRs and social neuroscience.

Therefore, in this article, we argue that IPS can be seen as a new interpretative proposal for neuroscientific findings surrounding personal growth that comes through the establishment of constructive interpersonal relationships. This proposal is an alternative understanding to the “autonomous-self theory” (AS), which understands the ‘self’ as a subject oriented toward productive activity and who seeks to improve personal cognitive abilities to build control. In addition, the “processual-self theory” (PS), though more dynamic with the inclusion of interpersonal relationships, continues to focus on the self as the only reference point for personal growth.

**FIGURE 1:**
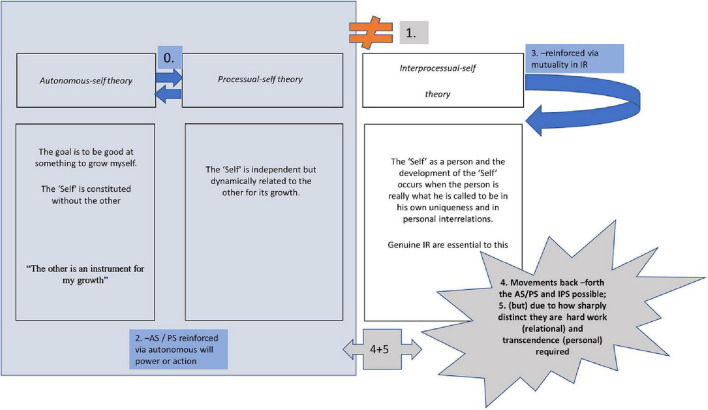
Graphic diagram on autonomous-self theory, processual-self theory and Inter Processual-self theory. IR, interpersonal relationship.

In Figure 1, we present a conceptual mapping of our proposal’s basic neuroscientific mechanisms. As portrayed, the AS and PS theories mutually reinforce each other and the neuroscience associated with them involves a cognitive switch between the two (0). On the other hand, the IPS theory is distinct and discontinuous from AS and PS in neuroscientific terms (1). AS/PS theories are reinforced via autonomous will power or action guided by a cognitive “mood” e.g., Cognitive/rational (AS) or more emotivist (PS) as pictured in (2). The IPS theory is reinforced via mutuality in IR (3). Movements back –forth between AS/PS and IPS are possible (4) and are up to the human beings involved in an IR, but, given their sharp distinction, hard work in IRs and personal transcendence are required (5). For a review of AS and PS theories see Akrivou et al. (2018, 2020).

## The Interpersonal Relationship as an Enhancer of Cognitive, Affective and Ethical-Moral Processes: a View From Neuroscience and Interprocessual-Self

Our proposal is strengthened by demonstrating the strong relationship between interpersonal relationships and cognitive, affective, and ethical processes. This section is thus framed by the social brain hypothesis perspective, which contains a personalist anthropology and manifests that the demands implied in living among social groups allow for the development of cognitive processes (Dunbar, 2003). This is in keeping with the primacy of Polo’s “personal radical” (2012) as a basic postulate for IPS; it assumes that the person is naturally concerned with growing in co-existence and relation with the other(s). By integrating interpersonal relationships and associated cognitive, affective and practical ethical-moral processes, we explore the direct and interactive effects of strong relationships on self-recognition, integration, and agency capacities (Kaplan et al., 2008; Powell et al., 2010; Orón Semper, 2015).

Before proceeding, it is worth noting that *cognitive/affective neuroscience* (CAN) began research by employing isolation paradigms (Becchio et al., 2010), understood as an experimental task that limits the social behavior of a participant to judging, categorizing, or inferring mental/emotional states from images or videos. Neuroimaging studies, which employ *functional magnetic resonance imaging* (fMRI), *computerized axial tomography* (CT), and *positron emission tomography* (PET), and neuropsychological studies have used this approach to identify and dissociate neural substrates and associated connectivity with cognitive domains involved in various areas of social cognition (Adolphs, 2010). Due to technical and methodological limitations, it only allows for unidirectional study of social-interaction (e.g., while reading a book or watching a movie) (García and Ibáñez, 2014), leaving aside person-to-person contact (e.g., a heated debate), which entails the most dynamic aspects of interaction (Bente et al., 2013). It should be noted that this review will focus on human functional magnetic resonance imaging studies and will not contemplate magnetoencephalography and neuroendocrine studies or with animals.

Currently, social CAN is exploring better methods for the study of person-to-person interactions (Liu and Pelowski, 2014). This exploration has led to the formalization of a new research area, namely *social interaction neuroscience* (SIN) (Hasson et al., 2012; Pfeiffer et al., 2013), which assumes that the neurobiological mechanisms involved in a person’s evaluation of social phenomena are different from the ones activated when people are involved in social interaction (García and Ibáñez, 2014). Thus, SIN promises findings that more adequately respond to the complexity of human relationships. This development has spurred new explanatory and interpretive frameworks and, in this context, IPS presents itself as an option given its understanding of interpersonal relationships, which it does not see as an instrument of individual development, but rather as a path toward development for each member of a relationship. In short, it understands the relationship itself as the goal.

However, SIN research development is recent, thus forcing us to review here findings from both *unidirectional* (CAN) and *interactive* (SIN) proposals that explain how interpersonal relationships can facilitate cognitive, affective and ethical-moral processes considered fundamental for self-knowledge, integration, agency and, in general, for personal-development (Pfeifer and Peake, 2012; Orón Semper et al., 2016; Schibli et al., 2017). Complexities arise that make it difficult to study the person in interpersonal relationships; associated processes are necessarily bio-directional at the interpersonal level and manifest as a more complicated multi-level phenomenon at the social level. Scientific observation has always struggled to adequality capture individual uniqueness and intimacy; for this reason, IPS’s theory of moral psychology does not neatly or comprehensively fit into social and cognitive/affective neuroscience, and instead requires a deeper understanding of those involved and their intimacy.

### The Interpersonal Relationship as an Enhancer of Cognitive Processes

Some studies suggest that social activities influence cognitive performance, arguing that interpersonal relationships allow for cognitive stimulation via the building of cognitive reserves that facilitate cognitive outcomes (Scarmeas and Stern, 2003). In this regard, neuroscience research with adult humans, the population to which we refer specifically in this review, is scarce, although there are studies that demonstrate an indirect benefit to speed and attention processing through the encouragement of health-oriented social behaviors like exercise among elderly people (Kelly et al., 2014). In addition, other studies identify social relationships as influential on speed processing, WM, visuospatial abilities, executive functions, and global cognition (Kelly et al., 2014). Finally, there is also evidence regarding the effects of stressful social situations on WM and in explicit and implicit memory (Luethi, 2009).

#### Working Memory

A relationship with another individual requires (simultaneous) representations of other-related and self-generated actions and the integration (online) of these representations in a perceptive process. For this to happen, the ability to simultaneously maintain and manipulate information in the short-term is fundamental, which corresponds to WM (Baddeley, 2012).

Working memory is supported by a fronto-parietal network that also includes hippocampal structures, amygdalae, and the cerebellum (Luis et al., 2015). These findings suggest that WM is engaged in planning, problem solving, reasoning (Suzuki et al., 2018) and intervenes in processing both emotional information and human movement (Shen et al., 2014). This function is important since the way an interlocutor’s movement is processed and interpreted can determine the other person’s possible response and behavior (Pavlova, 2012).

One unidirectional study found a positive correlation between WM’s ability to detect human movement and participants’ level of empathy (Gao et al., 2016). These results included interpretations like WM-processing of human movement contributes to our ability to empathize, meaning that the WM-buffer plays an important role in the transfer of social information in the course of perception. However, as a correlational study, an alternative interpretation based on IPS is possible. Namely, participants with high-levels of empathy possess greater WM-capacity for processing human movement and, in this way, their empathic behavior adapts to the specifics of the context where human movement is produced.

No clear body of research identifies if a significant relationship between human agents has a widespread impact on WM’s storage of interpersonal actions. Recently, Ding et al. (2017) implemented a more interactive paradigm using point-light displays with three experimental conditions, including (1) individuals-actions were presented in different locations, (2) sets of individuals-actions were presented in interaction, and (3) individual-action pairs were presented without interaction. Participants were asked to memorize individual actions and ignore social interactions. The results show that interactive actions were stored in WM as chunks that were not affected by the memory load. Thus, WM automatically and efficiently used a semantic knowledge strategy to store interactive actions that occurred in each trial (although researchers requested that participants consider social interaction as irrelevant). These results emphatically reflect the IPS proposal’s understanding of human action as the integration of cognitive, affective/intuitive, and practical, as well as ethical-moral, elements. Indeed, each participant implemented a semantic strategy for information storage that created personal singularity and intimacy.

#### Attention

An interpersonal encounter involves focusing one’s attention. People who interact not only pay attention to the other’s actions, but also to their own, simultaneously monitoring the context in which the encounter takes place. Some authors have defined this attention as foundational to action. On the one hand, there are attentional processes that involve a *dorsal network* (intraparietal sulcus and frontal ocular fields) and that are focused and directed toward the processing of an objective (top–down processing). On the other hand, a stimulus’s action (bottom–up processing) on the organism can elicit other attentional processes, activating a *ventral network* (ventrolateral prefrontal cortex and right temporo-parietal junction) (Corbetta and Shulman, 2002).

In this regard, we have not found attention studies that focus on improvement of interpersonal relationships. However, some studies have reported the importance of a new construct called “prioritized integrative attention or joint-attention,” which is understood as divided attention between one’s own actions and those of others while monitoring the wider context (Keller, 2008). For example, musicians combine selective attention on the chords that they have to produce (control of their own movements) together with joint-attention that focuses on monitoring the chords as a whole, which facilitates co-interpretation and the possibility that each member may adjust their actions when comparing the ideal sound to the one being produced (Repp and Keller, 2008; Keller et al., 2014).

Baron Cohen (1995) has proposed a model with four subprocesses that constitute joint-attention: (1) intention detection (fundamental to identifying the other as an agent); (2) a shared-attention mechanism (integrating one’s current perception of the self with a representation of the interpersonal relationship to build a global representation); (3) a theory of mind (ToM) mechanism (allowing for attribution of other people’s thoughts and intentions) and detection of eye direction (allowing for the establishment of an intentional relationship between agents through the direction of the gaze) (Saito et al., 2010). To date, the latter three aspects have received the most attention, connecting the neurosciences, research on the ToM, and their relationship with IPS.

In a relevant fMRI-study, participants had to interact contingently, through the gaze, with an animated character (apparently controlled by a human). The interaction consisted in (1) Guiding or (2) Being guided by the gaze of the virtual character, observing other objects present on the screen. The findings indicate that, when guiding, ventral striatum activation is associated with motivation to participate in interpersonal exchange. When being guided, anterior medial prefrontal cortex activation resulted from the coordination of perceptual and cognitive processes in relational-contexts (Schilbach et al., 2010). Another hyperscanning fMRI-study evaluated how visual-contact between pairs of adults provides a communicative bond that favors joint-attention processes, finding that the participants who synchronized their gaze activated the right inferior frontal gyrus (Saito et al., 2010). Similar results were found by Sadato (2016). These studies thus suggest that this brain region serves as a neural center for joint-attention in the exchange of intentions during eye-contact.

#### Cognitive Flexibility and Inhibitory Control

When the interpersonal relationship presents new challenges, its participants have to create new coping-strategies (cognitive flexibility) and, therefore, normal, daily responses are inhibited (traditionally called inhibition control). Mutual adaptation between people is the glue that allows for the development of joint action, but, in complex situations or where difference or novelty is interpreted as threatening, people may be predisposed to adapt to one another using different strategies (inhibition and cognitive flexibility strategies).

Cognitive flexibility is a construct that emerges from the correct functioning of executive functions and, therefore, involves cortical regions such as the ventro/dorsolateral prefrontal cortex, anterior cingulate, right anterior insula, premotor cortex, inferior and superior parietal cortices, inferior temporal cortex, occipital cortex, and subcortical structures like the thalamus and caudate nucleus (Dajani and Uddin, 2015). The ventrolateral prefrontal cortex is among the best-known neural substrates for inhibition control (Levy and Wagner, 2011), and corresponds to the anterior insular and inferior frontal junction involved in the detection of events relevant to behavior (Brass and Haggard, 2007; Tops and Boksem, 2011). With respect to the latter region, evidence that demonstrates a mediating role between dorsal/ventral attentional networks has also been found (Tamber-Rosenau et al., 2018).

To our knowledge, there are no reported hyperscanning neuroimaging studies involving inhibition and cognitive flexibility tasks in an intersubjective-exchange situation. However, several neurophysiological studies have provided findings that inform the relationship between interpersonal contact and the facilitation of performance in monitoring processes, or what has been called cognitive inhibition underlying flexible behavior aimed at objectives. A recent neurophysiological study explored how interpersonal touch in romantic couples affects the behavioral and neural processes that underlie flexible behavior directed toward goals. The results indicate that interpersonal contact improves intrapersonal neuronal monitoring processes that detect the need for cognitive control. In addition, this improved monitoring also indirectly predicts better inhibitory cognitive control (Saunders et al., 2018). Conversely, different studies have reported how conditions of deprivation in interpersonal relationships have an effect on inhibition and cognitive flexibility (mechanisms of cognitive control) (Leary et al., 2006). Lurquin et al. (2014) found that the incongruent trials of the Stroop test were accompanied by lower negativity of event-related potential (ERPs) N450. In addition, congruent trials presented greater negativity in the potential for the social exclusion of participant groups compared with control groups. These findings are not only in line with other studies (Xu et al., 2017), but also suggest that social exclusion has a deficit effect on cognitive inhibition and flexibility.

#### Episodic/Autobiographical Memory

Interpersonal relationships not only facilitate the storage of an interpersonal episode in the self but also favor the creation of AM of past interactions, which influences the social processing of future relationships. Over time, this information stored and subsequently integrated in the self enables the construction of a personal or group identity (Fivush, 2011).

Traditionally, AM is understood as a recall process of lived events to create a personal history (Conway and Pleydell-Pearce, 2000). The activation of the medial prefrontal cortex (Summerfield et al., 2009), medial posterior parietal cortex (MPPC) (precuneus, posterior cingulate, and retrosplenial/posterior cingulate cortices), and medial temporal lobe are fundamental for its optimal functioning (Spreng and Mar, 2012). On the other hand, episodic memory (EM) is defined as consciously recalling the events relevant to one’s personal past to project them onto future events (Wheeler, 2000). The type of “episode” influences EM’s neural substrates by involving other cortical areas of association, but, in general, activation of the hippocampus (the subiculum, the dentate gyrus, and the cornu ammonis regions), perirhinal cortex, parahippocampal cortex, and the prefrontal cortex are responsible for this process (Van Strien et al., 2009; Allen and Fortin, 2013). Other authors who implemented a relational paradigm episode (between a participant and another person) during a fMRI-study have found activation in the left precuneus and pre-supplementary motor areas, the right para-hippocampus, the bilateral fusiform gyrus, as well as insula activations. These brain areas are usually involved in processes like self-reflection, the understanding of others, and AM (Wade-Bohleber et al., 2019).

Overall, Wilbers et al. (2012) purport that AM is made up of semantic autobiographical memory (SAM) and episodic autobiographical memory (EAM). Specifically, the latter is considered the process involved in remembering an event, its location in time and space, and specific details with respect to other similar events (Piolino et al., 2003). Likewise, EAM is structured in a narrative manner, allowing for the construction of the autobiographical-self (Nelson, 2003).

However, EAM allows us to not only store information about ourselves, but also to establish flexible models to understand ourselves and our future relationships (Schacter et al., 2008; Eichenbaum and Fortin, 2009). This process is adequate for addressing the complexity of a novel or unknown social environment, decreasing the likelihood of a possible failure in social interaction, while also minimizing the likelihood of social rejection and isolation (Spreng and Mar, 2012).

One problem that seems to arise in determining the effect of interpersonal relationships on specific cognitive processes corresponds to the literature’s lack of clarity when defining the interpersonal indicators to be analyzed (Holt-Lunstad et al., 2010). For their part, Berkman et al. (2000) have proposed a conceptual framework to define participation in social activities, the amount of social resources available, and the perception of social support.

Studies have found that EM performs better in tasks that involve the processing of social information vs. non-social information (Reysen and Adair, 2008); in addition, closeness, i.e., a sense of common ground in a relationship, has been observed as allowing its interlocutors to share specific autobiographical memories (Beike et al., 2016). In fact, one meta-analysis linked subjective measures of social activities, social networks, social support, and measures related to social relationships with specific cognitive domains, including EM, finding a significant improvement effect when it comes to social support and composite measures of social relations on this type of memory. Some interpreted this result from the perspective of the impact that social support has on stress and its probable benefit in the performance of EM tasks (Kelly et al., 2017). Theoretically, this can also be explained from the perspective of the effect that social support can have on the development of episodic cognitive reserves that optimize cognitive performance by recruiting alternative brain networks to face more complex or novel future situations.

In short, in an interpersonal encounter, people share their interpretations of the world, their intentions for the world, and what happens to them; in other words, they share their intimacy. For each interacting person, this sharing implies thinking (cognitive representation) and feeling (affective) that one is familiar with the other. It involves a joint process between cognitive flexibility and WM since others’ representations are constructed from their attitudes, emotions or behaviors. Even though we all think we know ourselves (keeping a representation of what I am/what I share), as well as the other (keeping a representation of what the other is and shares), sometimes surprise is possible (when what I share or the other shares with me is not congruent with previous representation), which forces us to update old contents-representation and maintain those that are compatible, and involves long-term memory processes like autobiographical or semantic memory.

Faced with the different, novel, or strange situations that occur in interpersonal relationships (unexpected comments, out of context behavior, or constant change), or in the face of environmental change, dorsal and ventral attention networks and processes reported in the literature, like inhibition, must insert themselves in order to adequately address these situations. Therefore, the aforementioned cognitive processes are fundamental for personal development and facilitate social exchange processes.

Given that neuroscience research demonstrates a clear interrelation between individual cognitive processes and interpersonal relationships, our next step involves understanding this data in light of our chosen conceptual background, IPS, which assumes an interprocessual dimension.

### The Interpersonal Relationship as an Enhancer of Affective Processes

Some studies report the central role of affective dimensions for highly significant interpersonal experiences (Wade-Bohleber et al., 2019). For this reason, we will incorporate the IPS perspective, touching on things like empathy, physical contact and relational motivation, which are decisive both for navigating social environments and, therefore, for participants’ personal growth. We know that interpersonal relationships mediate many more emotional processes beyond those indicated in this article since human beings are complicated and rich organisms. For methodological simplicity, we have decided to focus on processes in which the relationship is fundamental, rather than on processes that imply individual actions. In this way, the processes considered here exclusively contemplate individual affective processes from an IPS perspective.

Emotional experience is configured in the first years of life not as reaction to stimuli, but rather relies on the quality of interpersonal relationships when experiencing the world. In this way, questions of who am I? who is the other? and what is the world? are answered simultaneously depending on interpersonal relationship quality (Winnicott, 1986; Lakovics, 1987; Orón Semper et al., 2020).

#### Physical Contact and Motivation

For authors such as Cascio et al. (2019), physical contact facilitates human development through social reward, the establishment of attachment (Bowlby, 1969; Schore, 2009), as well as strengthens cognitive processes, communication and emotional states throughout life. According to Hertenstein et al. (2007), physical contact between people allows them to experience states of happiness, security and motivation, which increases enjoyment and one’s disposition toward the tasks aimed at achieving objectives (Legg and Wilson, 2013).

The physical contact that takes place between people involves some measure of a reciprocal relationship (whether intimate or superficial). Several studies provide evidence of neural substrates in paradigms that involve sexual partners during intimate physical contact, as well as encounters between strangers (Hertenstein et al., 2009; Kreuder et al., 2017).

The neural substrates involved in physical contact at the peripheral level are related to the C-touch system (CTS) (low-threshold non-myelinated peripheral afferent fibers) that mediates passively received physical contact signals and overlaps with interlocutors’ affiliative reward and interception systems. At the cortical level, the somatosensory cortex (Ebisch et al., 2011; Gazzola et al., 2012), the posterior insular regions (Ebisch et al., 2014), the ventral striatum and anterior cingulate cortex (ACC) (Kreuder et al., 2017) are all involved in communication through physical contact.

Some studies have found that groups that experience physical contact are more cooperative, increasing the probability of competitive success in activities with other groups (Kraus et al., 2010; Coan and Sbarra, 2015). An interesting neurophysiological study found better outcomes in the Stroop test (color-word interference) in the context of physical contact; the authors see their results as indicating that interpersonal contact can induce intrinsic motivation by supporting autonomy and self-confidence (Lurquin et al., 2014). These results have been corroborated by studies from Legault and Inzlicht (2013) and Tjew-A-Sin et al. (2016), which, following neurophysiological measures, found that physical contact (simulated) led to higher Negativity related to Error (NRE) associated with high intrinsic motivation. In a similar vein, neuroimaging studies found increased perception of relief when subject to a painful stimulus and allowed physical contact with a romantic partner (Goldstein et al., 2018). There are also findings on the positive impact of physical contact in the relief of induced existential concerns (fear of death), especially in people with low self-esteem (Koole et al., 2014).

#### Empathy in Relationships

Empathy is born from the human need to perceive and understand the emotional states among interlocutors, with the aim of facilitating care, cooperation, and socialization (Cheng et al., 2014). Therefore, it results from the configuration of emotional and cognitive aspects that facilitate understanding of the other (Preston and de Waal, 2002). Authors such as De Waal (2008) suggest that empathy is energized by two systems: on the one hand, a system of basic emotional contagion associated with the lower frontal cortex (Seitz et al., 2008) and right insula (Wicker et al., 2003); it is in charge of recognizing the other’s emotional state and generating concern in the viewer. On the other hand, there is a more cognitive type of system that relates to the ventromedial prefrontal cortex (VMPFC) (Mitchell et al., 2006) and the temporoparietal junction (Samson et al., 2004). It mentalizes the emotional situation that conditions the other and tries to understand her perspective.

Neuroimaging studies that use paradigms of pain, disgust, and physical contact suggest that neuronal substrates that support interlocutors’ emotional and bodily states are similar to those involved in the ability to be empathetic with the other in an IR (Wicker et al., 2003; Keysers et al., 2004; Morrison et al., 2004). Activations in the sensorial motor cortex, medial and orbitofrontal cortex (OFC), the superior temporal cortex, the amygdala, cerebellum, the insular and in the ACC have traditionally been associated with pain discrimination (Apkarian et al., 2005; Emmert et al., 2014; Lu et al., 2016). However, studies have identified similar activations in the insular cortex and the ACC in paradigms that imply empathic pain felt on behalf of another person (Morrison et al., 2004). Thus, the biological resources of touch and empathy intersect with the entire social brain (Frith, 2007; Blakemore, 2008).

Similar activation areas in situations perceived in the first person and in the context of an empathic encounter lends itself to interpretation from the IPS perspective. Namely, IRs not only allow for the person to interpret her own reality, but also that of the other in real time. The latter allows for necessary adjustments to engage in behavior commensurate with the situation, which indicates that interpretation is not an action void of reality, but rather integrates the experiences of both participants in a relationship. The above is corroborated by studies that show how empathetic brain activation is modulated by the social relationships (affective attachment) that individuals share. For example, Singer et al. (2004) show how cooperative behaviors that nourish interpersonal bonds activate regions associated with empathy; conversely, selfish behaviors that compromise those bonds diminish or eliminate empathic brain responses.

### The Practical and Ethical-Moral Dimension of Interpersonal Relationships in the Context of Neuroscience

For IPS, the ethical and moral dimensions of the self are not additional to behavior, but rather are at the origin of human action. In our decisions, we decide who we want to be in relation to others, and this configures us. Ethics involves personal development in the midst of free and responsible action in face of the other, rather than of an ideal of behavior (Akrivou et al., 2018).

In neuroscience, morality has been studied in the context of tasks that allow for comparison between non-moral versus moral situations and by using moral dilemmas. Examples include the trolley paradigm and the footbridge dilemma (Navarrete et al., 2012; Özçiftci, 2017). These paradigms use complex situations that involve decision options between (1) death and/or (2) survival of more or less people; therein, the decisionmaker is presented with elements like degree of familiarity (Greene et al., 2004; Pujol et al., 2012).

The neural substrate is a complex process that links a large number of areas based on the implication of various cognitive and emotional subprocesses that support the establishment of moral judgment (Prehn et al., 2008; Pascual et al., 2013). At the subcortical level, this involves areas such as the hippocampus supporting the acquisition/recovery of fear conditioning (Gross and Gruart, 2009), the amygdala involved in moral learning and the evaluation of moral judgments (Mendez, 2006), the bilateral thalamus active in tasks that involve making decisions between a personal desire or a moral rule (Sommer et al., 2010), and the dorsal striatum in tasks that refer to altruistic punishment (De Quervain et al., 2004). They are constantly mentioned in the literature as important nodes of the networks associated with moral processing.

Likewise, the areas that intervene at the cortical level include the VMPFC, the emotional mediator in moral processing (Young and Koenigs, 2007), the OFC, which participates in the online representation of reward and punishment (Shenhav and Greene, 2010), the DLPFC plays a fundamentally executive role in the analysis of situations that require knowledge based on rules (Prehn et al., 2008) and in predictions based on social norms with inferences about the intention to deceive (Hu et al., 2015). In the ToM and in self-referential tasks (Frith, 2001), the ACC is involved in the monitoring of moral conflicts (Greene et al., 2004), the temporoparietal union (TPJ) plays a key role in moral intuition and in the attribution of beliefs to others during moral processing (Harada et al., 2009), and the temporal superior sulcus is associated with the establishment of inferences about others’ beliefs and intentions (Allison et al., 2000).

Some authors suggest that the default mode network (DMN) regions, like the medial prefrontal cortex (MPFC), the posterior cingulate cortex (PCC), precuneus, and the inferior parietal lobe (IPL), are not only involved in self-referential and autobiographical processes but also related to the processing of moral information (Han, 2016). In fact, experimental paradigms that compare admiration via an ethical-moral procedure versus others’ physical conditions evidence the participation of DMN areas therein (Englander et al., 2012; Immordino-Yang et al., 2012) and in tasks that involve prosocial behavior, moral emotions, and moral cognition (Moll et al., 2007; Reniers et al., 2012; Sevinc and Nathan Spreng, 2014).

All this demonstrates that the moral-ethical aspect of personal action is a truly complicated process and, in order to understand it, we need a deeper knowledge of each actor and situation on an almost case-by-case analysis. It also reveals, in line with IPS theory, why the ethical aspects of action are not a separate component, but rather are part of the unifying and binding aspects of the self and action. We know that both the intuitive-heuristic (affective) and analytical (cognitive) reasoning processes are crucial for detecting conflicts and avoiding errors in decision-making in situations with ethical-moral implications (De Neys and Glumicic, 2008). However, to date, research on the interaction between these two processes in ethical-moral situations has failed to yield conclusive data. In this regard, Greene and Haidt (2002) propose three possible theoretical explanations of their interaction: (1) The *social intuitionist theory* considers moral judgment an automatic process that is influenced by social and cultural conditions (Haidt, 2001); (2) The *cognitive control and conflict theory* postulates that the brain activation associated with cognitive aspects results in different processing in areas in the brain related to emotion (Greene et al., 2004); and (3) The *cognitive and emotional integration theory* purports that specific cortical-subcortical loops that organize social cognition, emotion, and motivation support ethical-moral processing (Gottfried, 1999).

Interprocessual-self presents an alternative, affirming that moral judgment arises when defining the type of interpersonal relationship. It is not about absorbing social principles, nor about individual cognitive or cognitive-emotional acts. For example, the classic trolley paradigm reveals that familiarity radically influences the decision of who will die. In this sense, the key is seeing the other as someone rather than just as a number, that is, the type of relationship in question guides the kind of moral judgment used. In brief, IPS and IRs are not just about emotion and cognition separately, but rather are about the need to co-develop with the other (intimacy), while developing one’s singularity and achieving higher self-understanding.

As we can see, there is ground for further theory development and empirical validation linking IPS and SIN within the practical ethical-moral aspects of neuroscience. Such a move would aim to expand beyond conflict detection and error-free decision-making processes (linked to intuitive-heuristic and analytical/cognitive control and conflict theories and to ones related to the emotions in neuroscience) in the direction of expanding our understanding of how different persons resolve ethical dilemmas. This resolution includes cognition/attachment and empathic/affective processing while acting with a commitment to co-develop personhood within a context of (greater or lesser) intimacy, enabling the self and others to grow. It assumes that our understanding of moral situations is much more personal and intimately felt than we currently account for.

## Self-Knowledge, Integration, and Agency: An Explanation From the Interprocessual-Self Perspective

These ideas, ranging from the description of cognitive, emotional, and moral processes, are articulated to facilitate more complex processes such as self-knowledge, integration, and agency. We aim to demonstrate and clarify that all human aspects must be understood on equal footing with brain activity, i.e., as an integrated system and not as an addition of analytical dimensions.

To achieve this, we present three processes that involve the participation of the previous processes (cognitive, emotional, and moral) in existential moments within the framework of IPS and neuroscience. Such moments include understanding oneself as a person (*self-knowledge*) capable of integration, how life and the other relate to oneself (*integration*), and how that relationship influences my ability to do or create (*agency*).

### Self-Knowledge and Reflected Self-Appraisal Processes Facilitated by Interpersonal Relationships

The significance of IPS is best revealed at the level of personal identity, but, since the construction of personal identity is hard to study from neuroscience, we will focus on self-knowledge, which is part of personal identity and also possible to study within the neuroscience research paradigm.

Self-knowledge and the reflexive self-evaluation process are important for cognitive, affective, ethical-moral, and social functioning. Traditionally, the reflexive self-appraisal process is defined as the internalization of others’ (perceived) opinions, contributing to the consolidation of self-knowledge (Ochsner et al., 2005). According to Nelson et al. (2013), the latter process can be subdivided into two levels, as follows: the pre-reflexive level that groups information associated with bodily, self, and self-agency processing and that is associated with insular activation (Wittmann, 2015), and the reflexive-self level, which involves narrative or autobiographical sub-processes associated with midline brain structures (Zaytseva et al., 2014).

In particular, neural substrates from the self-knowledge process have frequently been associated with activity originating in the ventral medial prefrontal cortex (vMPFC), the precuneus, and the posterior cingulate in the posterior medial parietal cortex (Wagner, 2012; Wagner et al., 2012; D’Argembeau, 2013). On the other hand, reflexive self-appraisals involve the dorsal MPFC (dMPFC), the temporal-parietal junction (TPJ), the superior posterior temporal sulcus (pSTS), and the temporal poles (Gallagher and Frith, 2003; Saxe and Kanwisher, 2003). Therefore, according to Denny et al. (2012) in their fMRI meta-analytic study, the MPFC contains a gradient of ventral to dorsal functional specialization. That is, ventral regions are associated with self-knowledge processes, while dorsal regions are responsible for processing information about other people. In fact, some authors have interpreted this to indicate DMN specialization, when it is actually involved in self-referential processing (Van Buuren et al., 2010).

These areas are also related to closely identified concepts and personal beliefs (Orón Semper, 2014). Psychology, self-recognition, self-awareness, self-esteem, self-conception, capacity for agency, among other constructs, can all be included under the wide umbrella of the self (Alicke and Sedikides, 2011). The construction of the self implies identity construction, which contains personal (that which distinguishes the person), as well as social elements (attachments, relationships between groups, and roles) (Erikson, 1968). Thus, each of us perceives ourselves as a unified, distinct and enduring entity (Pfeifer et al., 2007) in relation to others.

In the process of developing personal and social identity, the information that feeds these processes is mutually shared. For some authors, self-knowledge allows for consolidation of personal identity; it involves not only cognitive capacities (a personal component) but also other components (especially social ones). In turn, self-knowledge is processed by the former component, which mediates the latter; here the loop emerges, feeding back into the consolidation of identity (Harter et al., 1999; Forrester, 2001). In fact, self-knowledge is considered a result of the internalization or incorporation of perspectives that other individuals hold about us in our self-concept, as well as others’ behavioral patterns (Harter and Leahy, 2018). This interactive process is supported by cognitive processes such as attention, WM, episodic-autobiographical memory, cognitive flexibility and inhibition, as well as socio-affective processes such as physical contact, motivation or empathy (as discussed previously); these are all particularly important for self-awareness and reflexive self-appraisal, and, at the same time, are fundamental for personal growth (Pfeifer and Peake, 2012).

The foregoing arguments allow us to understand why IPS assumes that the self is essentially revealed through the IRs in which it engages. It emphasizes the profound role of personal intimacy for self-knowledge and implies that neither an isolated-self nor a static-self can be understood. At the same time, IPS maintains the relevance of IRs and one’s own authorship because it is not just a synthesis of relationships, but rather an active determination of relational quality while achieving personal (virtuous) growth.

### Integration as a Process of Increasing Self-Knowledge Facilitated by Interpersonal Relationships

As mentioned earlier, several studies in neuroscience, such as those from Lieberman et al. (2004) and D’Argembeau et al. (2005), have suggested greater activation in the medial prefrontal cortex (MPFC) and the MPPC with the execution of self-knowledge enhancing tasks. However, other studies have reported activations in these same areas when making inferences about others (Schmitz et al., 2004; Ochsner et al., 2005). Overall, processes involving self-knowledge and knowledge of others— especially exploring others’ similarity to one’s self (Benoit et al., 2010)— share MPFC and MPPC activations (Mitchell et al., 2005; Pfeifer et al., 2007; Jenkins et al., 2008).

As a processing and association center for these two information types, these regions evidence that IRs empower integration due to the enriching effect of bidirectional information flows (from self-knowledge to knowledge of the other and vice versa), which, in turn, translates into creative ideas for meeting life’s challenges. Likewise, these areas also facilitate long-term integration of interpersonal experience, allowing for the creation of social knowledge (Spreng and Mar, 2012). Such knowledge is embodied in the mutual fostering of personal and interpersonal skills, and in the flexibility to respond to a novel or complex situation. Based on the above, IPS understands integration as an ongoing, natural capacity that is supported by a two-way feedback mechanism: (1) The interpersonal relationship itself (inter) and (2) Self-knowledge and lived experience (processual). Thus, integration is a process of mutually increasing self-understanding and growth based on interaction with others; it rejects an understanding of relationships as mere instruments for individual growth (Akrivou and Orón, 2016).

Given this, IPS mainly rests on systemic integration of the person and, therein, on a real and sincere dialogue involving interpersonal relationships. Such dialogue comes from the interiority of the person and involves the integration of cognitive, emotional, and ethical/moral dimensions. IPS differentiates realities without separating them from the personal and the social realms and is a more fluid model since its central reference point is the person and relational development.

### Agency

The agency capacity is defined as the experience of actions themselves. It has also been defined as the ability to control one’s own actions and, through them, influence environmental conditions (Frith, 2014; Khalighinejad and Haggard, 2016). Therefore, this capacity is fundamental for the achievement of goal-oriented behaviors and for instrumental action, which are relevant for humans when changing the surrounding environment (Moll et al., 2007). The existing literature frequently associates voluntary action with a reconstructed inference to the monitoring of actions themselves and the results obtained (subjective experience or sense of agency), with brain processes related to the preparation of action, or with a combination of the two (the monitoring of actions and their results or interpretation starting from when action is prepared) (Khalighinejad and Haggard, 2016).

Some authors have suggested that agency depends on motor mechanisms and conscious processes. Recently, Frith (2014) published new data that integrate the two previous models. According to this theory, a sense of agency depends on the processes involved in the predictive control of action at a conscious level: the attenuation of sensory feedback specific to our own actions. This attenuation depends on the accuracy of the comparison between the predicted state and the actual state. In addition, a sense of agency also implies the management of the social framework, which usually involves human interaction.

Some neuroscientists have suggested that voluntary action merits neuronal activity in motor and cognitive areas located in the frontal cortex medial (Fried et al., 2011). Other results have associated a sense of agency with preparatory volitional signals linked to fronto-parietal areas (Desmurget et al., 2009; Fried et al., 2011), which subsequently activate voluntary motor commands associated with the primary motor cortex (Sherrington, 1906), which is considered a “final common path.”

However, a meta-analytical and connectivity study (which assumes that sense of agency and motor intention must occur at different times in the generation of voluntary actions) (Seghezzi et al., 2019) has reported that sense of agency is supported by a so-called “self-agency network” constituted by the SMA regions (posterior medial frontal cortex), the posterior insula, the occipital lobe and cerebellum. Functionally, this is responsible for simple motor control, sensorimotor processes, and the elicitation of manifest movement (Showers and Lauer, 1961; Kim et al., 2010; Kurth et al., 2010). On the other hand, it also identifies another network that is more associated with the motor intention or an “intentionality network.” In this network, the middle cingulate and pre-supplementary motor regions (rostral medial frontal cortex), anterior insula, and parietal cortex participate; they are conceptualized as an executive level system that is involved in alternating motor plans, changing tasks, acquiring new motor skills, motor selection, response selection, conflict monitoring, self-referential, cognitive and emotional functions (Mayer et al., 2007; Nachev et al., 2008; Singer et al., 2009; De Baene and Brass, 2013). Finally, together with these networks with dissociated functions, there is a shared network made up of the meso-frontal and prefrontal regions, the middle insula, and the subcortical structures.

The IPS model is relevant for understanding the agency because, instead of just focusing on behavior or cognitive elements, IPS understands agency inside of the complexity of human action, integrating all the dimensions of human action within a social network. From the IPS perspective, action integrates every human dimension, defining the quality of our personal relationships and, at the same time, our identity.

## Socialization as a Fundamental Variable of Human Action

The analytical view tends to see each human aspect as an independent construction. Throughout this argument, however, it is becoming clearer that neuroscientific research points to the fact that all human aspects must be understood in an integrated and systemic way since they relate to brain activity. Sociability’s impact on the study of brain functioning urges us to consider human beings as a system.

Sociability is of such importance that it has been incorporated into fMRI experiments that involve simultaneous hyper-scans of two people in interaction even though, as mentioned previously, most neuroscience experiments are designed for individual scans (Liu and Pelowski, 2014). The human brain is formed by interaction with other people and it requires, above all, medial areas of the prefrontal cortex, and synchronization is achieved thanks to the activity of the lateral frontoparietal network (Sänger et al., 2011). According to Clark-Polner and Clark (2014) the social realm impacts thoughts, perception, feelings, beliefs, and empathy. In addition, for these authors, the human social character is not sufficiently taken into consideration in neuroscience trials, since it is often wrongly assumed that our social character only intervenes when it comes to explicitly social activities.

The affective base built in childhood guarantees subsequent personal development and affects perception, cognition, learning, emotion, and physical development. For humans, the physical treatment that a child receives from her mother explains how she accesses the world. In the absence of a loving triangle between mother–child-the world, the child interprets the world as an insecure place and ends up associating stimulus with aggression (Cyrulnik, 2005). This is so because the mother presents the world to the child: maternal sensitivity modulates the relationship between the environment and the child’s genetics, affecting her behavior (Zhang et al., 2014). The mother-child relationship occurs very early; for example, at just 34 weeks *in utero*, a fetus learns his mother’s speech tone and speed. The child’s stress mechanism responds to changes in the mother’s voice, which can be evaluated through heart pulsations (Krueger and Garvan, 2014). This reveals that personal relationships are not just another personal dynamic, but rather the natural dynamic of personal growth (Orón Semper et al., 2020).

Studies of the social brain reveal not only the presence of the social realm in personal life but also that the whole brain is social (Blakemore, 2008). While a clearly social task can be designed to study the ToM, this dimension is ubiquitous; the social and non-social are inseparable. It is often said in neuroscience that choices between material goods are made by way of codifying reward valuation and that, for their part, social valuations are processed differently, in a way that focuses on representing the self and others. In reality, both mechanisms are shared, making up a single mechanism (Ruff and Fehr, 2014).

Other studies confirm similar activations, in this case of the striatum, both for the social and for the non-social (Báez-Mendoza and Schultz, 2013). The biological, the social, the emotional, and the cultural shape our various development trajectories, and the mother–child relationship affects the configuration of the amygdala, medial cortex, orbitofrontal, accumbens, and insula (Silk et al., 2014). Therefore, sociality marks the rhythm of maturation itself and is present from the beginning of the human experience. These reactive levels reflect some innate and other cultural aspects, revealing that human beings’ social nature is inscribed in biology. And said biology is social biology. For example, in an electroencephalography study, 12 high school students in biology class were exposed to two experimental conditions: (1) a face-to-face class and (2) a video-recorded class. Both groups were subject to evaluation. The findings show that students with greater social proximity to the teacher presented greater brain synchronicity between them; additionally, a correlation was found between teacher/student proximity and students’ retention capacity. Thus, aspects of socialization, in this case proximity, reflect brain-to-brain synchronization and can predict cognitive performance (Bevilacqua et al., 2018). Similar results were described by Dikker et al. (2017),Bevilacqua et al. (2018), and Pöysä et al. (2018).

In the same vein, young children only learn a language if it is a means to interpersonal encounter; exposure to stimuli without interpersonal experience does not lead to learning (Kuhl et al., 2003). In this way, the social relationship is not a simple modulator of language; rather a social relationship triggers, enables, opens up, and motivates language acquisition (Kuhl, 2014). To this, we can add that all the great psychological lines of human development agree that the appearance of language sparked human potentiality. Thus, at the base of all human development is the experience of interpersonal encounters.

### Mutual Trust and Human Intent

The psychoanalyst Kohut (1977) proposes that human beings are born in trust (which is then confirmed or denied) and Erikson (1968), also a psychoanalyst, locates human beings’ first challenge in the trust-mistrust binomial. Thus trust, a highly relevant element in the IPS proposal, places the reception of an interpersonal encounter at the base of human development. As a process rooted in human biology, it seems clear that human biology is social biology. For example, with just 50 ms of exposure, we already know whether the other inspires confidence or not, and this assessment correlates with activity in the amygdala (Freeman et al., 2014); after 100 ms, evaluation of the confidence aroused by the presence of the other is complete (Vernon et al., 2014). Surprisingly, these studies are conducted such that an explicit trust assessment is not necessary, showing how natural relationships and interpersonal encounters are. Indeed, they are constituent of human beings, as IPS maintains.

### Cooperation Between People

Some studies have recently begun to focus on changes in perspective-taking between people. This socio-cognitive process allows us to recognize and appreciate empathy, another person’s point of view, and whether or not the interlocutors share anything (Healey and Grossman, 2018). It is subdivided into cognitive components (the ability to infer the other’s thoughts) and affective ones (the ability to infer the other’s feelings or emotions). Both involve regions such as the temporoparietal junction, the precuneus, and the temporal poles, but they differ when it comes to taking affective perspective involving regions within the limbic system and the basal ganglia, as well as the VMPFC. This is contrary to taking cognitive perspective, which is associated with the dorsolateral and dorsomedial prefrontal cortex (Hynes et al., 2006; Sebastian et al., 2012; Corradi-Dell’Acqua et al., 2014). Taking perspective is a process intimately linked to empathy, and specifically to subprocesses like internal simulation and adoption of mental states between interlocutors, which is fundamental for appropriate social interaction.

But the ability to take on the other’s perspective is not enough to definitively establish the need for IPS. When Rizzolatti discovered mirror neurons in monkeys, he claimed to have found the biological basis of empathy. But the issue is much more complex. Without going into the whole debate, it is worth noting that Rizzolatti himself discovered that activation is not automatic, but rather involves how reality is valued. Monkeys have utilitarian knowledge of one another and only utility activates mirror neurons (Rizzolatti et al., 1988). That is, monkeys use subject-subject relationships for their own interest. On the other hand, for human beings, another person’s movement is a valuable thing to pay attention to regardless of utility (Fadiga et al., 1995), a quality that better fits the IPS model, which puts the interpersonal relationship in the center.

Likewise, interpersonal relationships not only demand empathy, but also the capacity for reciprocity, trust, and cooperation. These conditions can naturally imply investment and small individual losses on the part of participants, to later facilitate collective gains. In addition, they imply the ability to take responsibility for one’s own acts (agency) (Anderson, 2003; Frith, 2014; Steffel and Williams, 2018). Some studies report a shift from a “self-agency” state to a personal-relational “I-thou-agency,” which also involves the wider social “we-agency,” since individuals are connected in personal and broader relations that imply the development of collective action and involve cooperation among participants (Pacherie, 2013; Dewey et al., 2014).

Functional magnetic resonance imaging studies on empathy and cooperation include Singer et al.’s (2006) implementation of a sequentially iterated prisoner’s dilemma, thus presenting the possibility of (1) playing in a more cooperative or trustworthy way by sending other participants greater amounts of money, allowing all players to gain and diminishing non-cooperative behavior/distrust. (2) This implies that the other player’s valuation of these actions is fair or unfair respectively. (3) Inside the fMRI scanner (after determining participants’ taste or distaste for other players’ way of playing), the researchers determined how empathic participants were when observing small electric shocks for infringements when applied to (1) themselves, (2) players seen as fair, and (3) players seen as unfair. The results showed greater brain responses in regions associated with reward processing (ventral striatum, nucleus accumbens, and left frontal orbital cortex) by assuming that pain experienced by unfair players is just punishment. These results are seen as showing that interpersonal relationships valued as fair allow for mutual reward and empathic behavior. An alternative interpretation, starting from IPS, argues that empathy and cooperation facilitate the creation and maintenance of interpersonal bonds, which contrasts with the selfish behavior that puts said bonds at risk by spurring on the diminution or elimination of brain activity associated with empathic behavior.

On the other hand, in a study that linked four experiments on trust among people, Bahrami et al. (2010, 2016) sought to answer the question of how two different individuals can combine the processing of visual sensory signals through social interaction. In other words, is it justifiable to think that “two heads are better than one?” To answer this question, they implemented a perceptual decision task applied to pairs of individuals. Their results showed how fluid communication and trust between people benefits decision-making in a paradigm of perceptual sensitivity, but only when observers are equally sensitive. These findings corroborate IPS’ assumptions, sustaining those participants who enter into a relationship in equal conditions mutually contribute to enhanced performance (a situation that contrasts with the relationships implied in social loafing or interpersonal competition, where performance at the group level does not surpass individual performance).

To conclude and with regard to the limitations of the present study, it is necessary to state that the literature contemplated in this review has been limited to functional magnetic resonance studies with adult humans and did not contemplate a developmental approach that included the effect of interpersonal relationships at different stages of the life cycle. However, we believe that although the evolutionary perspective of interpersonal relationships is interesting, this approach exceeds the claim of the article, which was to propose a new interpretation of the data thrown from the neurosciences on the importance of interpersonal relationships.

Another limitation is that fMRI studies tend to have relatively small sample sizes. An attempt was made to overcome this situation by trying to relate and contrast the results of different studies with different sample sizes to point to the value of interpersonal relationships. However, this is still a limitation compared to the assumptions that each study can show and can have an effect on what is contemplated in the present study.

On the other hand, and as mentioned previously, some of the included studies present paradigms oriented to interpersonal relationships but measured by acquiring data from a single participant interacting with a computer, likewise, other studies, due to technical limitations, show little ecological value in the face of a real situation, which means that its conclusions have to be assumed with caution. To overcome this limitation, we presented both single and multiscan studies.

## Conclusion, Limitations, and Future Research

This review introduces the interprocessual-self theory as a novel and useful theoretical proposal for the experimental design and interpretation of neuroscientific findings on personal growth. We suggest based on theoretical and neuroscience-based empirical evidence that there is empirical evidence in support of that interpersonal relations play a key role for human growth and the development of essential personal growth aspects including integration, the self and self-knowledge, and the capacity for (ethical) agency. To do so we introduce a new theory “Interprocessual-self” which is a new theory in this regard and draws from a rich albeit systematic compilation of empirical studies in neurosciences to show evidence for the claims of the theory with some further support from anthropological and ethical sources.

Throughout this article, we highlighted how interpersonal relationships have a significant impact on cognitive, emotional, and ethical-moral processes. To demonstrate this influence, we included a detailed conceptualization and description of the neural bases that support the interrelation of these processes and growth-inducing relationships. In terms of evidence from neuroscience, we first describe related research in support of the idea that personal virtue in IRs and relevant cognitive, emotional, and ethical-moral processes are not independent but related aspects of personal growth; while we include descriptors, research, and concepts from neural bases of this we demonstrate how interpersonal relationships allow all persons involved in an IR to grow and to relate ethically. As a second step to providing evidence concerning the new theoretical model, we draw from neuroscience empirical studies on the self and namely: self-knowledge, integration, and agency. We also evidence from studies in neuroscience relevant to the totality of psychological processes that stimulate inner exploration of the self about the other (relationally) showing that these can be understood from IPS theory; as a final step, we demonstrate how the theory involves the integration of two levels of neuroscientific relational processes, namely the interpersonal one and the social one. Traditionally, this research has been done with an understanding of the person as an acting individual whose relationships with the other mediate her activity. This model no longer does justice to contemporary advances in neuroscience, which opens up the possibility of considering an alternative model that sees relationships as constitutive of human beings and human action. In summary, this understanding between theory and neuroscientific evidence supports the relevant role of interpersonal relationships in the development of essential aspects associated with personal growth, including self-knowledge, integration, and the capacity for agency. These psychological processes can be improved upon and nourished by the interprocessual-self, which stimulates internal exploration of the self to the other.

We aspire to be a novel and relevant contribution supported by rich scientific evidence via neuroscience and wider research from fields that inform the latter to help us make progress in understanding personal growth as a matter of IR. Neuroscience and related data as a source of evidence in light of understanding the human being(s) are valid for a rigorous methodology. But of course, it is a method that is incomplete and needs further other sources when it comes to attempting to capture and understand the human beings and interpersonal relational growth: beyond neuroscience, a richer wider qualitative methodology, original biographical and other personal historical information and triangulation of qualitative and quantitative methods would provide a deeper source of data. Also to understand the human being(s), requires a proper theoretical reliance on philosophical anthropology, moral philosophy, and psychology to allow a fuller presentation of a rich anthropological basis to demonstrate what makes up the uniqueness of being, acting, and growing as human being involves something much more profound, which requires too, a methodology that opens the way for a theory of the person that responds to the concerns of philosophy and philosophical anthropology from many disciplines and methods (Orón Semper, 2015; Polo, 2015),

However, we hope this work will open a dialogue to cross-validate and triangulate findings and evidence with other methods, especially qualitative and less analytical, which points to both a limitation of this current research and also invites for future research directions and potentialities. As noted, to fully study the human beings’ ethical-cognitive-affective and action processing in IR is of course something that is complicated and requires a more profound anthropological basis enabling a richer and more profound understanding of what it means to be human. This is not in the scope of this current research.

## Data Availability Statement

The original contributions presented in the study are included in the article/supplementary material, further inquiries can be directed to the corresponding author/s.

## Author Contributions

EL and JO: conceptualization, formal analysis, methodology, validation, writing–original draft, and writing–review and editing. KA: conceptualization, data curation, methodology, validation, writing–original draft, and writing–review and editing. EB-M: formal analysis, methodology, validation, writing–original draft, and writing–review and editing. GS: writing–original draft and writing–review and editing. All authors: contributed to the article and approved the submitted version.

## Conflict of Interest

The authors declare that the research was conducted in the absence of any commercial or financial relationships that could be construed as a potential conflict of interest.

## Publisher’s Note

All claims expressed in this article are solely those of the authors and do not necessarily represent those of their affiliated organizations, or those of the publisher, the editors and the reviewers. Any product that may be evaluated in this article, or claim that may be made by its manufacturer, is not guaranteed or endorsed by the publisher.

## References

[B1] Adolphs R. (2010). Conceptual challenges and directions for social neuroscience. *Neuron* 65 752–767. 10.1016/j.neuron.2010.03.006 20346753 PMC2887730

[B2] Akrivou K. Orón J. V. (2016). “Two kinds of human integrity: towards the ethics of inter-processual self (IPS),” in *Challenges of Capitalism for Virtue and the Common Good: Interdisciplinary Perspectives*, eds Akrivou K. Sison A. J. G. (London: Edward Elgar), 221–253. 10.4337/9781784717919.00019

[B3] Akrivou K. Orón J. V. Scalzo G. (2018). *The Inter-Processual Self: Towards a Personalist Virtue Ethics Proposal for Human Agency.* Cambridge: Cambridge Scholars Publishing.

[B4] Akrivou K. Scalzo G. Orón J. V. (2020). “The moral psychology of practical wisdom for business and management,” in *Handbook of Practical Wisdom in Business and Management. International Handbooks in Business Ethics*, eds Schwartz B. Bernacchio C. González-Cantón C. Robson A. (Berlin: Springer), 10.1007/978-3-030-00140-7_15-1

[B5] Alicke M. D. Sedikides C. (Eds.) (2011). *Handbook of Self-Enhancement and Self-Protection.* New York, NY: The Guilford Press.

[B6] Allen T. A. Fortin N. J. (2013). The evolution of episodic memory. *Proc. Natl. Acad. Sci. U.S.A.* 110 (Suppl_2) 10379–10386. 10.1073/pnas.1301199110 23754432 PMC3690604

[B7] Allison T. Puce A. McCarthy G. (2000). Social perception from visual cues: role of the STS region. *Trends Cogn Sci* 4 267–278. 10.1016/S1364-6613(00)01501-1 10859571

[B8] Anderson C. J. (2003). The psychology of doing nothing: forms of decision avoidance result from reason and emotion. *Psychol. Bullet.* 129, 139–167. 10.1037/0033-2909.129.1.139 12555797

[B9] Apkarian A. V. Bushnell M. C. Treede R. D. Zubieta J. K. (2005). Human brain mechanisms of pain perception and regulation in health and disease. *Eur. J. Pain* 9 463–484. 10.1016/j.ejpain.2004.11.001 15979027

[B10] Arancibia M. D. (2018). “The notion of freedomaccording to the transcendental anthropology of leonardo polo,” in *Psychiatry and Neuroscience Update*, eds Gargiulo P. Mesones Arroyo H. (Cham: Springer), 11–22. 10.1007/978-3-319-95360-1_2

[B11] Baddeley A. (2012). Working memory: theories, models, and controversies. *Annu. Rev. Psychol.* 63 1–29. 10.1146/annurev-psych-120710-100422 21961947

[B12] Báez-Mendoza R. Schultz W. (2013). The role of the striatum in social behavior. *Front. Neurosci.* 7:233. 10.3389/fnins.2013.00233 24339801 PMC3857563

[B13] Bahrami B. Olsen K. Latham P. E. Roepstorff A. Rees G. Frith C. D. (2010). Optimally interacting minds. *Science* 329 1081–1085. 10.1126/science.1185718 20798320 PMC3371582

[B14] Bahrami B. Olsen K. Latham P. E. Roepstorff A. Rees G. Frith C. D. (2016). “Optimally interacting minds,” in *Discovering the Social Mind: Selected Works of Christopher*, ed. Frith D. (London: Routledge). 10.4324/9781315630502

[B15] Baron Cohen S. (1995). *Mindblindness: Essay on Autism and the Theory of Mind, Learning. Development and Conceptual Change.* Cambridge: MIT Press.

[B16] Becchio C. Sartori L. Castiello U. (2010). Toward you: the social side of actions. *Curr. Direct. Psychol. Sci.* 19 183–188. 10.1177/0963721410370131

[B17] Beike D. R. Brandon N. R. Cole H. E. (2016). Is sharing specific autobiographical memories a distinct form of self-disclosure? *J. Exp. Psychol.* 145 434–450. 10.1037/xge0000143 26974311

[B18] Benoit R. G. Gilbert S. J. Volle E. Burgess P. W. (2010). When I think about me and simulate you: medial rostral prefrontal cortex and self-referential processes. *NeuroImage* 50 1340–1349. 10.1016/j.neuroimage.2009.12.091 20045478

[B19] Bente G. Reddy V. Timmermans B. Schlicht T. Costall A. Schilbach L. (2013). Toward a second-person neuroscience. *Behav. Brain Sci.* 36 393–414. 10.1017/s0140525x12000660 23883742

[B20] Berkman L. F. Glass T. Brissette I. Seeman T. E. (2000). From social integration to health: durkheim in the new millennium. *Soc. Sci. Med.* 51 843–857. 10.1016/S0277-9536(00)00065-4 10972429

[B21] Bevilacqua D. Davidesco I. Wan L. Chaloner K. Rowland J. Ding M. (2018). Brain-to-brain synchrony and learning outcomes vary by student-teacher dynamics: evidence from a real-world classroom electroencephalography study. *J. Cogn. Neurosci.* 31 401–411. 10.1162/jocn_a_01274 29708820

[B22] Blakemore S. J. (2008). The social brain in adolescence. *Nat. Rev. Neurosci.* 9 267–277. 10.1038/nrn2353 18354399

[B23] Bowlby J. (1969). Attachment and loss: attachment. *Attachment* 1 470–478. 10.1177/000306518403200125

[B24] Brass M. Haggard P. (2007). To do or not to do: the neural signature of self-control. *J. Neurosci.* 27 9141–9145. 10.1523/jneurosci.0924-07.2007 17715350 PMC6672190

[B25] Cascio C. J. Moore D. McGlone F. (2019). Social touch and human development. *Dev. Cogn. Neurosci.* 35 5–11. 10.1016/j.dcn.2018.04.009 29731417 PMC6968965

[B26] Cheng Y. Chen C. Decety J. (2014). An EEG/ERP investigation of the development of empathy in early and middle childhood. *Dev. Cogn. Neurosci.* 10 160–169. 10.1016/j.dcn.2014.08.012 25261920 PMC6987874

[B27] Clark-Polner E. Clark M. S. (2014). Understanding and accounting for relational context is critical for social neuroscience. *Front. Hum. Neurosci.* 8:127. 10.3389/fnhum.2014.00127 24723868 PMC3971189

[B28] Coan J. A. Sbarra D. A. (2015). Social baseline theory: the social regulation of risk and effort. *Curr. Opin. Psychol.* 1 87–91. 10.1016/j.copsyc.2014.12.021 25825706 PMC4375548

[B29] Conway M. A. Pleydell-Pearce C. W. (2000). The construction of autobiographical memories in the self-memory system. *Psychol. Rev.* 107 261–288. 10.1037/0033-295X.107.2.261 10789197

[B30] Corbetta M. Shulman G. L. (2002). Control of goal-directed and stimulus-driven attention in the brain. *Nat. Rev. Neurosci.* 3 201–215. 10.1038/nrn755 11994752

[B31] Corradi-Dell’Acqua C. Hofstetter C. Vuilleumier P. (2014). Cognitive and affective theory of mind share the same local patterns of activity in posterior temporal but not medial prefrontal cortex. *Soc. Cogn. Affect. Neurosci.* 9 1175–1184. 10.1093/scan/nst097 23770622 PMC4127022

[B32] Cyrulnik B. (2005). Ethology and the biological correlates of mood. *Dial. Clin. Neurosci.* 7 217–221. 10.31887/DCNS.2005.7.3/bcyrulnik PMC3181736 16156380

[B33] Dajani D. R. Uddin L. Q. (2015). Demystifying cognitive flexibility: implications for clinical and developmental neuroscience. *Trends Neurosci.* 38 571–578. 10.1016/j.tins.2015.07.003 26343956 PMC5414037

[B34] D’Argembeau A. (2013). On the role of the ventromedial prefrontal cortex in self-processing: the valuation hypothesis. *Front. Hum. Neurosci.* 7:372. 10.3389/fnhum.2013.00372 23847521 PMC3707083

[B35] D’Argembeau A. Collette F. Van Der Linden M. Laureys S. Del Fiore G. Degueldre C. (2005). Self-referential reflective activity and its relationship with rest: a PET study. *NeuroImage* 25 616–624. 10.1016/j.neuroimage.2004.11.048 15784441

[B36] De Baene W. Brass M. (2013). Switch probability context (in)sensitivity within the cognitive control network. *NeuroImage* 77 207–214. 10.1016/j.neuroimage.2013.03.057 23567890

[B37] De Neys W. Glumicic T. (2008). Conflict monitoring in dual process theories of thinking. *Cognition* 106 1248–1299. 10.1016/j.cognition.2007.06.002 17631876

[B38] De Quervain D. J. F. Fischbacher U. Treyer V. Schellhammer M. Schnyder U. Buck A. (2004). The neural basis of altruistic punishment. *Science* 305 1254–1258. 10.1126/science.1100735 15333831

[B39] De Waal F. B. M. (2008). Putting the altruism back into altruism: the evolution of empathy. *Annu. Rev. Psychol.* 59 279–300. 10.1146/annurev.psych.59.103006.093625 17550343

[B40] Denny B. T. Kober H. Wager T. D. Ochsner K. N. (2012). A meta-analysis of functional neuroimaging studies of self- and other judgments reveals a spatial gradient for mentalizing in medial prefrontal cortex. *J. Cogn. Neurosci.* 24 1742–1752. 10.1162/jocn_a_00233 22452556 PMC3806720

[B41] Desmurget M. Reilly K. T. Richard N. Szathmari A. Mottolese C. Sirigu A. (2009). Movement intention after parietal cortex stimulation in humans. *Science* 324, 811–813. 10.1126/science.1169896 19423830

[B42] Dewey J. A. Pacherie E. Knoblich G. (2014). The phenomenology of controlling a moving object with another person. *Cognition* 132 383–397. 10.1016/j.cognition.2014.05.002 24879353

[B43] Dikker S. Wan L. Davidesco I. Kaggen L. Oostrik M. McClintock J. (2017). Brain-to-brain synchrony tracks real-world dynamic group interactions in the classroom. *Curr. Biol.* 27 1375–1380. 10.1016/j.cub.2017.04.002 28457867

[B44] Ding X. Gao Z. Shen M. (2017). Two equals one: two human actions during social interaction are grouped as one unit in working memory. *Psychol. Sci.* 28 1311–1320. 10.1177/0956797617707318 28719763

[B45] Dunbar R. I. M. (2003). The social brain: mind, language, and society in evolutionary perspective. *Annu. Rev. Anthropol.* 32 163–181. 10.1146/annurev.anthro.32.061002.093158

[B46] Ebisch S. J. Ferri F. Gallese V. (2014). Touching moments: desire modulates the neural anticipation of active romantic caress. *Front. Behav. Neurosci.* 8:60. 10.3389/fnbeh.2014.00060 PMC3937548 24616676

[B47] Ebisch S. J. H. Ferri F. Salone A. Perrucci M. G. D’Amico L. Ferro F. M. (2011). Differential involvement of somatosensory and interoceptive cortices during the observation of affective touch. *J. Cogn. Neurosci.* 23 1808–1822. 10.1162/jocn.2010.21551 20666597

[B48] Eichenbaum H. Fortin N. J. (2009). The neurobiology of memory based predictions. *Philos. Trans. R. Soc. B* 364 1183–1191. 10.1098/rstb.2008.0306 19527999 PMC2666706

[B49] Emmert K. Breimhorst M. Bauermann T. Birklein F. Van De Ville D. Haller S. (2014). Comparison of anterior cingulate vs. insular cortex as targets for real-time fMRI regulation during pain stimulation. *Front. Behav. Neurosci.* 8:350. 10.3389/fnbeh.2014.00350 25346666 PMC4191436

[B50] Englander Z. A. Haidt J. Morris J. P. (2012). Neural basis of moral elevation demonstrated through inter-subject synchronization of cortical activity during free-viewing. *PLoS One* 7:e39384. 10.1371/journal.pone.0039384 22745745 PMC3379986

[B51] Erikson E. H. (1968). *Identity, Youth, and Crisis.* New York, NY: W. W. Norton Company.

[B52] Fadiga L. Fogassi L. Pavesi G. Rizzolatti G. (1995). Motor facilitation during action observation: a magnetic stimulation study. *J. Neurophysiol.* 73 2129–2617. 10.1152/jn.1995.73.6.2608 7666169

[B53] Fernández González M. J. (2019). At the heart of virtue growth: ‘self-of-virtue’ and ‘virtue identity. *Estudios Sobre Educ.* 36 9–29. 10.15581/004.36.9-29

[B54] Fivush R. (2011). Development of Autobiographical Memory. *Annu. Rev. Psychol.* 62 559–582. 20636128 10.1146/annurev.psych.121208.131702

[B55] Forrester M. A. (2001). The embedding of the self in early interaction. *Infant Child Dev.* 10 189–202. 10.1002/icd.278

[B56] Freeman J. B. Stolier R. M. Ingbretsen Z. A. Hehman E. A. (2014). Amygdala responsivity to high-level social information from unseen faces. *J. Neurosci.* 34 10573–10581. 10.1523/JNEUROSCI.5063-13.2014 25100591 PMC6802589

[B57] Fried I. Mukamel R. Kreiman G. (2011). Internally generated preactivation of single neurons in human medial frontal cortex predicts volition. *Neuron* 69, 548–562. 10.1016/j.neuron.2010.11.045 21315264 PMC3052770

[B58] Frith C. D. (2007). The social brain? *Philos. Trans. R. Soc. B* 362 671–678. 10.1098/rstb.2006.2003 17255010 PMC1919402

[B59] Frith C. D. (2014). Action, agency and responsibility. *Neuropsychologia* 55 137–142. 10.1016/j.neuropsychologia.2013.09.007 24036357

[B60] Frith U. (2001). Mind blindness and the brain in autism. *Neuron* 32 969–979. 10.1016/S0896-6273(01)00552-9 11754830

[B61] Gallagher H. L. Frith C. D. (2003). Functional imaging of “theory of mind.”. *Trends Cogn. Sci.* 7 77–83. 10.1016/s1364-6613(02)00025-6 12584026

[B62] Gao Z. Ye T. Shen M. Perry A. (2016). Working memory capacity of biological movements predicts empathy traits. *Psychon. Bull. Rev.* 23 468–475. 10.3758/s13423-015-0896-2 26174575

[B63] García A. M. Ibáñez A. (2014). Two-person neuroscience and naturalistic social communication: the role of language and linguistic variables in brain-coupling research. *Front. Psychiatry* 5:124. 10.3389/fpsyt.2014.00124 25249986 PMC4155792

[B64] Gazzola V. Spezio M. L. Etzel J. A. Castelli F. Adolphs R. Keysers C. (2012). Primary somatosensory cortex discriminates affective significance in social touch. *Proc. Natl. Acad. Sci. U.S.A.* 109 E1657–E1666. 10.1073/pnas.1113211109 22665808 PMC3382530

[B65] Goldstein P. Weissman-Fogel I. Dumas G. Shamay-Tsoory S. G. (2018). Brain-to-brain coupling during handholding is associated with pain reduction. *Proc. Natl. Acad. Sci. U.S.A.* 115 E2528–E2537. 10.1073/pnas.1703643115 29483250 PMC5856497

[B66] González R. C. (2018). El hábito de sabiduría y el carácter de además. *Stud. Poliana* 20 33–63. 10.15581/013.20.33-63

[B67] González R. C. (2019). El hombre en la antropología trascendental de Polo. *Stud. Poliana* 21 29–53. 10.15581/013.21.29-53

[B68] Gottfried K. (1999). Moral calculus and the bomb. *Nature* 401:117. 10.1038/43571

[B69] Greene J. Haidt J. (2002). How (and where) does moral judgment work? *Trends Cogn. Sci.* 6 517–523. 10.1016/s1364-6613(02)02011-9 12475712

[B70] Greene J. D. Nystrom L. E. Engell A. D. Darley J. M. Cohen J. D. (2004). The neural bases of cognitive conflict and control in moral judgment. *Neuron* 44 389–400. 10.1016/j.neuron.2004.09.027 15473975

[B71] Gross C. Gruart A. (2009). S.23.02 Role of hippocampus in the cognitive control of anxiety. *Eur. Neuropsychopharmacol.* 19:S210. 10.1016/s0924-977x(09)70284-6

[B72] Haidt J. (2001). The emotional dog and its rational tail: a social intuitionist approach to moral judgment. *Psychol. Rev.* 108 814–834. 10.1037/0033-295X.108.4.814 11699120

[B73] Han H. (2016). How can neuroscience contribute to moral philosophy, psychology and education based on Aristotelian virtue ethics? *Int. J. Ethics Educ.* 1 201–217. 10.1007/s40889-016-0016-9

[B74] Harada T. Itakura S. Xu F. Lee K. Nakashita S. Saito D. N. (2009). Neural correlates of the judgment of lying: a functional magnetic resonance imaging study. *Neurosci. Res.* 63 24–34. 10.1016/j.neures.2008.09.010 18992288

[B75] Harter S. Bresnick S. Bouchey H. Whitesell N. (1999). “The construction of the self: a developmental perspective,” in *Proccedings of the Integrative Processes and Socialization Early to Middle Childhood*, (Beijing), 27–71. 10.1109/BigMM.2015.82

[B76] Harter S. Leahy R. L. (2018). The construction of the self: a developmental perspective. *J. Cogn. Psychother.* 15 383–384. 10.1891/0889-8391.15.4.383 11261958

[B77] Hasson U. Ghazanfar A. A. Galantucci B. Garrod S. Keysers C. (2012). Brain-to-brain coupling: a mechanism for creating and sharing a social world. *Trends Cogn. Sci.* 16 114–121. 10.1016/j.tics.2011.12.007 22221820 PMC3269540

[B78] Healey M. L. Grossman M. (2018). Cognitive and affective perspective-taking: evidence for shared and dissociable anatomical substrates. *Front. Neurol.* 25:491. 10.3389/fneur.2018.00491 29988515 PMC6026651

[B79] Hertenstein M. J. Holmes R. McCullough M. Keltner D. (2009). The communication of emotion via touch. *Emotion* 9 566–573. 10.1037/a0016108 19653781

[B80] Hertenstein M. J. Verkamp J. M. Kerestes A. M. Holmes R. M. (2007). The communicative functions of touch in humans, nonhuman primates, and rats: a review and synthesis of the empirical research. *Genet. Soc. Gen. Psychol. Monogr.* 132 5–94. 10.3200/MONO.132.1.5-94 17345871

[B81] Holt-Lunstad J. Smith T. B. Layton J. B. (2010). Social relationships and mortality risk: a meta-analytic review. *PLoS Medicine* 7:e1000316. 10.1371/journal.pmed.1000316 20668659 PMC2910600

[B82] Hu X. Pornpattananangkul N. Nusslock R. (2015). Executive control- and reward-related neural processes associated with the opportunity to engage in voluntary dishonest moral decision making. *Cogn. Affect. Behav. Neurosci.* 15 475–491. 10.3758/s13415-015-0336-9 25645507 PMC5133289

[B83] Hynes C. A. Baird A. A. Grafton S. T. (2006). Differential role of the orbital frontal lobe in emotional versus cognitive perspective-taking. *Neuropsychologia* 44 374–383. 10.1016/j.neuropsychologia.2005.06.011 16112148

[B84] Immordino-Yang M. H. Christodoulou J. A. Singh V. (2012). Rest is not idleness: implications of the brain’s default mode for human development and education. *Perspect. Psychol. Sci.* 7 352–364. 10.1177/1745691612447308 26168472

[B85] Jenkins A. C. Macrae C. N. Mitchell J. P. (2008). Repetition suppression of ventromedial prefrontal activity during judgments of self and others. *Proc. Natl. Acad. Sci. U.S.A.* 105 4507–4512. 10.1073/pnas.0708785105 18347338 PMC2393803

[B86] Kaplan J. T. Aziz-Zadeh L. Uddin L. Q. Iacoboni M. (2008). The self across the senses: an fMRI study of self-face and self-voice recognition. *Soc. Cogn. Affect. Neurosci.* 3 218–223. 10.1093/scan/nsn014 19015113 PMC2566765

[B87] Keller P. E. (2008). Joint action in music performance. *Enacting Intersubjectivity* 10 205–221.

[B88] Keller P. E. Novembre G. Hove M. J. (2014). Rhythm in joint action: psychological and neurophysiological mechanisms for real-time interpersonal coordination. *Philos. Trans. R. Soc. B* 369:20130394. 10.1098/rstb.2013.0394 25385772 PMC4240961

[B89] Kelly M. E. Duff H. Kelly S. McHugh Power J. E. Brennan S. Lawlor B. A. (2017). The impact of social activities, social networks, social support and social relationships on the cognitive functioning of healthy older adults: a systematic review. *Syst. Rev.* 6:259. 10.1186/s13643-017-0632-2 29258596 PMC5735742

[B90] Kelly M. E. Loughrey D. Lawlor B. A. Robertson I. H. Walsh C. Brennan S. (2014). The impact of exercise on the cognitive functioning of healthy older adults: a systematic review and meta-analysis. *Ageing Res. Rev.* 16 12–31. 10.1016/j.arr.2014.05.002 24862109

[B91] Keysers C. Wicker B. Gazzola V. Anton J.-L. Fogassi L. Gallese V. (2004). A touching sight: SII/PV activation during the observation and experience of touch. *Neuron* 42, 335–346. 10.1016/S0896-6273(04)00156-4 15091347

[B92] Khalighinejad N. Haggard P. (2016). Extending experiences of voluntary action by association. *Proc. Natl. Acad. Sci. U.S.A.* 113 8867–8872. 10.1073/pnas.1521223113 27436902 PMC4978305

[B93] Kim J. H. Lee J. M. Jo H. J. Kim S. H. Lee J. H. Kim S. T. (2010). Defining functional SMA and pre-SMA subregions in human MFC using resting state fMRI: functional connectivity-based parcellation method. *NeuroImage* 49 2375–2386. 10.1016/j.neuroimage.2009.10.016 19837176 PMC2819173

[B94] Kohut H. (1977). *The Termination of the Analysis of Narcissistic Personality Disorders. In The Restoration of the Self.* Chicago, IL: University of Chicago Press, 1–62.

[B95] Koole S. L. Tjew A. Sin M. Schneider I. K. (2014). Embodied terror management: interpersonal touch alleviates existential concerns among individuals with low self-esteem. *Psychol. Sci.* 25 30–37. 10.1177/0956797613483478 24190907

[B96] Kraus M. W. Huang C. Keltner D. (2010). *Running Head: Touch, Cooperation, and Performance: An Ethological Study of the NBA. Touch, Cooperation, and Performance 1, University of California, B.* 1–20. 10.1037/a0019382 21038960

[B97] Kreuder A. K. Scheele D. Wassermann L. Wollseifer M. Stoffel-Wagner B. Lee M. R. (2017). How the brain codes intimacy: the neurobiological substrates of romantic touch. *Hum. Brain Mapp.* 38 4525–4534. 10.1002/hbm.23679 28580708 PMC6867116

[B98] Krueger C. Garvan C. (2014). Emergence and retention of learning in early fetal development. *Infant Behav. Dev.* 37 162–173. 10.1016/j.infbeh.2013.12.007 24548971

[B99] Kuhl P. K. (2014). Early language learning and the social brain. *Cold Spring Harb. Symp. Quantitative Biol.* 79 211–220. 10.1101/sqb.2014.79.024802 25943768

[B100] Kuhl P. K. Tsao F. M. Liu H. M. (2003). Foreign-language experience in infancy: effects of short-term exposure and social interaction on phonetic learning. *Proc. Natl. Acad. Sci. U.S.A.* 100 9096–9101. 10.1073/pnas.1532872100 12861072 PMC166444

[B101] Kurth F. Eickhoff S. B. Schleicher A. Hoemke L. Zilles K. Amunts K. (2010). Cytoarchitecture and probabilistic maps of the human posterior insular cortex. *Cereb. Cortex (New York, N.Y.: 1991)* 20 1448–1461. 10.1093/cercor/bhp208 19822572 PMC2871375

[B102] Lakovics M. (1987). Home is where we start from: essays by a psychoanalyst. *Am. J. Psychother.* 41 145–146. 10.1176/appi.psychotherapy.1987.41.1.145

[B103] Leary M. R. Twenge J. M. Quinlivan E. (2006). Interpersonal rejection as a determinant of anger and aggression. *Pers. Soc. Psychol. Rev.* 10 111–132. 10.1207/s15327957pspr1002_2 16768650

[B104] Legault L. Inzlicht M. (2013). Self-determination, self-regulation, and the brain: autonomy improves performance by enhancing neuroaffective responsiveness to self-regulation failure. *J. Pers. Soc. Psychol.* 105 123–138. 10.1037/a0030426 23106250

[B105] Legg A. M. Wilson J. H. (2013). Instructor touch enhanced college students’ evaluations. *Soc. Psychol. Educ.* 16 317–327. 10.1007/s11218-012-9207-1

[B106] Levy B. J. Wagner A. D. (2011). Cognitive control and right ventrolateral prefrontal cortex: reflexive reorienting, motor inhibition, and action updating. *Ann. N. Y. Acad. Sci.* 1224 40–62. 10.1111/j.1749-6632.2011.05958.x 21486295 PMC3079823

[B107] Lieberman M. D. Jarcho J. M. Satpute A. B. (2004). Evidence-based and intuition-based self-knowledge: an fMRI study. *J. Pers. Soc. Psychol.* 87, 421–435. 10.1037/0022-3514.87.4.421 15491269

[B108] Liu T. Pelowski M. (2014). Clarifying the interaction types in two-person neuroscience research. *Front. Hum. Neurosci.* 8:276. 10.3389/fnhum.2014.00276 24817848 PMC4012218

[B109] Lu C. Yang T. Zhao H. Zhang M. Meng F. Fu H. (2016). Insular cortex is critical for the perception, modulation, and chronification of pain. *Neurosci. Bull.* 32 191–201. 10.1007/s12264-016-0016-y 26898298 PMC5563738

[B110] Luethi M. (2009). Stress effects on working memory, explicit memory, and implicit memory for neutral and emotional stimuli in healthy men. *Front. Behav. Neurosci.* 2:5. 10.3389/neuro.08.005.2008 19169362 PMC2628592

[B111] Luis E. O. Arrondo G. Vidorreta M. Martinez M. Loayza F. Fernández-Seara M. A. (2015). Successful working memory processes and cerebellum in an elderly sample: a neuropsychological and fMRI study. *PLoS One* 10:e0131536. 10.1371/journal.pone.0131536 26132286 PMC4488500

[B112] Lurquin J. H. McFadden S. L. Harbke C. R. (2014). An electrophysiological investigation of the effects of social rejection on self control. *J. Soc. Psychol.* 154 186–197. 10.1080/00224545.2014.881768 24873022

[B113] Mayer J. S. Bittner R. A. Nikolić D. Bledowski C. Goebel R. Linden D. E. J. (2007). Common neural substrates for visual working memory and attention. *NeuroImage* 36 441–453. 10.1016/j.neuroimage.2007.03.007 17462914

[B114] Mendez M. F. (2006). What frontotemporal dementia reveals about the neurobiological basis of morality. *Med. Hypoth.* 67 411–418. 10.1016/j.mehy.2006.01.048 16540253

[B115] Mitchell J. P. Banaji M. R. Macrae C. N. (2005). The link between social cognition and self-referential thought in the medial prefrontal cortex. *J. Cogn. Neurosc.* 17 1306–1315. 10.1162/0898929055002418 16197685

[B116] Mitchell J. P. Macrae C. N. Banaji M. R. (2006). Dissociable medial prefrontal contributions to judgments of similar and dissimilar others. *Neuron* 50 655–663. 10.1016/j.neuron.2006.03.040 16701214

[B117] Moll J. de Oliveira-Souza R. Garrido G. J. Bramati I. E. Caparelli-Daquer E. M. A. Paiva M. L. M. F. (2007). The self as a moral agent: linking the neural bases of social agency and moral sensitivity. *Soc. Neurosci.* 2 336–352. 10.1080/17470910701392024 18633822

[B118] Morrison I. Lloyd D. Di Pellegrino G. Roberts N. (2004). Vicarious responses to pain in anterior cingulate cortex: is empathy a multisensory issue? *Cogn. Affect. Behav. Neurosci.* 4 270–278. 10.3758/CABN.4.2.270 15460933

[B119] Nachev P. Kennard C. Husain M. (2008). Functional role of the supplementary and pre-supplementary motor areas. *Nat. Rev. Neurosci.* 9 856–869. 10.1038/nrn2478 18843271

[B120] Navarrete C. D. McDonald M. M. Mott M. L. Asher B. (2012). Virtual morality: emotion and action in a simulated three-dimensional “trolley problem”. *Emotion* 12, 364–370. 10.1037/a0025561 22103331

[B121] Nelson B. Thompson A. Yung A. R. (2013). Not all first-episode psychosis is the same: preliminary evidence of greater basic self-disturbance in schizophrenia spectrum cases. *Early Intervent. Psychiatry* 7 200–204. 10.1111/j.1751-7893.2012.00381.x 22759705

[B122] Nelson K. (2003). Self and social functions: individual autobiographical memory and collective narrative. *Memory* 11 125–136. 10.1080/741938203 12820826

[B123] Ochsner K. N. Beer J. S. Robertson E. R. Cooper J. C. Gabrieli J. D. E. Kihsltrom J. F. (2005). The neural correlates of direct and reflected self-knowledge. *NeuroImage* 28 797–814. 10.1016/j.neuroimage.2005.06.069 16290016

[B124] Orón Semper J. V. (2014). Neuroscience and Faith: the belief system as a venue of interdisciplinary meeting. *Neurosci. Faith* 2 213–270. 10.12775/SetF.2014.021

[B125] Orón Semper J. V. (2015). Leonardo Polo’s integrative dynamic as a philosophical framework for understanding neuroscience. *J. Polian Stud.* 2 109–133.

[B126] Orón Semper J. V. (2020). *Encuentro Interprocesual. Por un Mundo para el Crecimiento Interpersonal.* Madrid: ICCE.

[B127] Orón Semper J. V. Akrivou K. Scalzo G. (2019). Educational implications that arise from differing models of human development and their repercussions on social innovation. *Front. Educ.* 4:139. 10.3389/feduc.2019.00139

[B128] Orón Semper J. V. Murillo J. I. Bernacer J. (2016). Adolescent emotional maturation through divergent models of brain organization. *Front. Psychol.* 7:1263. 10.3389/fpsyg.2016.01263 27602012 PMC4993867

[B129] Orón Semper J. V. Navarro-Rubio S. Luis E. O. (2020). Emotional education for personal growth in the early years. *J. Theor. Philos. Psychol.* 41 115–130. 10.1037/teo0000150

[B130] Özçiftci V. M. (2017). The trolley problem. *Turkish J. Bioethics* 3, 226–227. 10.5505/tjob.2016.33042

[B131] Pacherie E. (2013). Intentional joint agency: shared intention lite. *Synthese* 190 1817–1839. 10.1007/s11229-013-0263-7

[B132] Pascual L. Rodrigues P. Gallardo-Pujol D. (2013). How does morality work in the brain? A functional and structural perspective of moral behavior. *Front. Integr. Neurosci.* 7:65. 10.3389/fnint.2013.00065 24062650 PMC3770908

[B133] Pavlova M. A. (2012). Biological motion processing as a hallmark of social cognition. *Cereb. Cortex* 22 981–995. 10.1093/cercor/bhr156 21775676

[B134] Pfeifer J. H. Lieberman M. D. Dapretto M. (2007). “i know you are but what am i?!”: neural bases of self- and social knowledge retrieval in children and adults. *J. Cogn. Neurosci.* 19 1323–1337. 10.1162/jocn.2007.19.8.1323 17651006 PMC3407805

[B135] Pfeifer J. H. Peake S. J. (2012). Self-development: integrating cognitive, socioemotional, and neuroimaging perspectives. *Dev. Cogn. Neurosci.* 2 55–69. 10.1016/j.dcn.2011.07.012 22682728 PMC6987679

[B136] Pfeiffer U. J. Timmermans B. Vogeley K. Frith C. D. Schilbach L. (2013). Towards a neuroscience of social interaction. *Front. Hum. Neurosci.* 7:22. 10.3389/fnhum.2013.00022 23378836 PMC3561599

[B137] Piolino P. Desgranges B. Belliard S. Matuszewski V. Lalevée C. De La Sayette V. D. (2003). Autobiographical memory and autonoetic consciousness: triple dissociation in neurodegenerative diseases. *Brain* 126 2203–2219. 10.1093/brain/awg222 12821510

[B138] Polo L. (1971). La cuestión de la esencia extramental. *Anuario Filosófico* 4 273–308.

[B139] Polo L. (1999). La amistad en Aristóteles. *Anuario Filosófico* 32 477–485. 10.15581/009.32.2.477-485

[B140] Polo L. (2015). *Why a Transcendental Anthropology? South Bend.* Chicago, IL: Leonardo Polo Institute of Philosophy Press.

[B141] Polo L. Corazón R. (2005). *Lo Radical y la Libertad [The Radical and the Freedom]. Cuadernos de Anuario Filosófico, 179.* Pamplona: Servicio de publicaciones de la Universidad de Navarra.

[B142] Powell L. J. Macrae C. N. Cloutier J. Metcalfe J. Mitchell J. P. (2010). Dissociable neural substrates for agentic versus conceptual representations of self. *J. Cogn. Neurosci.* 22 2186–2197. 10.1162/jocn.2009.21368 19925182

[B143] Pöysä S. Vasalampi K. Muotka J. Lerkkanen M. K. Poikkeus A. M. Nurmi J. E. (2018). Teacher-student interaction and lower secondary school students’ situational engagement. *Br. J. Educ. Psychol.* 89 374–392. 10.1111/bjep.12244 30252125

[B144] Prehn K. Wartenburger I. Mériau K. Scheibe C. Goodenough O. R. Villringer A. (2008). Individual differences in moral judgment competence influence neural correlates of socio-normative judgments. *Soc. Cogn. Affect. Neurosci.* 3 33–46. 10.1093/scan/nsm037 19015093 PMC2569820

[B145] Preston S. D. de Waal F. B. M. (2002). Empathy: its ultimate and proximate bases. *Behav. Brain Sci.* 25 1–20. 10.1017/S0140525X02000018 12625087

[B146] Pujol J. Batalla I. Contreras-Rodríguez O. Harrison B. J. Pera V. Hernández-Ribas R. (2012). Breakdown in the brain network subserving moral judgment in criminal psychopathy. *Soc. Cogn. Affect. Neurosci.* 7 917–923. 10.1093/scan/nsr075 22037688 PMC3501707

[B147] Reniers R. L. E. P. Corcoran R. Völlm B. A. Mashru A. Howard R. Liddle P. F. (2012). Moral decision-making, ToM, empathy and the default mode network. *Biol. Psychol.* 90 202–210. 10.1016/j.biopsycho.2012.03.009 22459338

[B148] Repp B. H. Keller P. E. (2008). Sensorimotor synchronization with adaptively timed sequences. *Hum. Move. Sci.* 27 423–456. 10.1016/j.humov.2008.02.016 18405989

[B149] Reysen M. B. Adair S. A. (2008). Social processing improves recall performance. *Psychon. Bull. Rev.* 15 197–201. 10.3758/PBR.15.1.197 18605503

[B150] Rizzolatti G. Camarda R. Fogassi L. Gentilucci M. Luppino G. Matelli M. (1988). Functional organization of inferior area 6 in the macaque monkey. *Exp. Brain Res.* 71 491–507. 10.1007/bf00248742 3416965

[B151] Ruff C. C. Fehr E. (2014). The neurobiology of rewards and values in social decision making. *Nat. Rev. Neurosci.* 15 549–562. 10.1038/nrn3776 24986556

[B152] Sadato N. (2016). Neuroimaging approach to the functional neuroanatomy: from human brain mapping of the single brain towards network-network analysis of real-time social interaction as “Two-in-One” system using hyper-scanning fMRI. *Jpn J. Neurosurg.* 25 421–426. 10.7887/jcns.25.421

[B153] Saito D. N. Tanabe H. C. Izuma K. Hayashi M. J. Morito Y. Komeda H. (2010). Stay tuned”: inter-individual neural synchronization during mutual gaze and joint attention. *Front. Integr. Neurosci.* 4:127. 10.3389/fnint.2010.00127 21119770 PMC2990457

[B154] Samson D. Apperly I. A. Chiavarino C. Humphreys G. W. (2004). Left temporoparietal junction is necessary for representing someone else’s belief. *Nate. Neurosci.* 7 499–500. 10.1038/nn1223 15077111

[B155] Sänger J. Lindenberger U. Müller V. (2011). Interactive brains, social minds. *Commun. Integr. Biol.* 4 655–663. 10.4161/cib.17934 22448303 PMC3306325

[B156] Saunders B. Riesel A. Klawohn J. Inzlicht M. (2018). Interpersonal touch enhances cognitive control: a neurophysiological investigation. *J. Exp. Psychol.* 147 1066–1077. 10.1037/xge0000412 29565607

[B157] Saxe R. Kanwisher N. (2003). People thinking about thinking people. *NeuroImage* 19 1835–1842. 10.1016/S1053-8119(03)00230-1 12948738

[B158] Scarmeas N. Stern Y. (2003). Cognitive reserve and lifestyle. *J. Clin. Exp. Neuropsychol.* 25 625–633. 10.1076/jcen.25.5.625.14576 12815500 PMC3024591

[B159] Schacter D. L. Addis D. R. Buckner R. L. (2008). Episodic simulation of future events: concepts, data, and applications. *Ann. N. Y. Acad. Sci.* 1124 39–60. 10.1196/annals.1440.001 18400923

[B160] Schibli K. Wong K. Hedayati N. D’Angiulli A. (2017). Attending, learning, and socioeconomic disadvantage: developmental cognitive and social neuroscience of resilience and vulnerability. *Ann. N. Y. Acad. Sci.* 1396 19–38. 10.1111/nyas.13369 28548461

[B161] Schilbach L. Wilms M. Eickhoff S. B. Romanzetti S. Tepest R. Bente G. (2010). Minds made for sharing: initiating joint attention recruits reward-related neurocircuitry. *J. Cogn. Neurosci.* 22 2702–2715. 10.1162/jocn.2009.21401 19929761

[B162] Schmitz T. W. Kawahara-Baccus T. N. Johnson S. C. (2004). Metacognitive evaluation, self-relevance, and the right prefrontal cortex. *NeuroImage* 22 941–947. 10.1016/j.neuroimage.2004.02.018 15193625

[B163] Schore A. N. (2009). Relational trauma and the developing right brain: an interface of psychoanalytic self psychology and neuroscience. *Ann. N. Y. Acad. Sci.* 1159 189–203. 10.1111/j.1749-6632.2009.04474.x 19379241

[B164] Sebastian C. L. Fontaine N. M. G. Bird G. Blakemore S. J. De brito S. A. Mccrory E. J. P. (2012). Neural processing associated with cognitive and affective theory of mind in adolescents and adults. *Soc. Cogn. Affect. Neurosci.* 7 53–63. 10.1093/scan/nsr023 21467048 PMC3252629

[B165] Seghezzi S. Zirone E. Paulesu E. Zapparoli L. (2019). The brain in (Willed) action: a meta-analytical comparison of imaging studies on motor intentionality and sense of agency. *Front. Psychol.* 10:804. 10.3389/fpsyg.2019.00804 31031676 PMC6473038

[B166] Seitz R. J. Schäfer R. Scherfeld D. Friederichs S. Popp K. Wittsack H. J. (2008). Valuating other people’s emotional face expression: a combined functional magnetic resonance imaging and electroencephalography study. *Neuroscience* 152 713–722. 10.1016/j.neuroscience.2007.10.066 18313858

[B167] Sevinc G. Nathan Spreng R. (2014). Contextual and perceptual brain processes underlying moral cognition: a quantitative meta-analysis of moral reasoning and moral emotions. *PLoS One* 9 e0087427. 10.1371/journal.pone.0087427 24503959 PMC3913597

[B168] Shen M. Gao Z. Ding X. Zhou B. Huang X. (2014). Holding biological motion information in working memory. *J. Exp. Psychol.* 40 1332–1345. 10.1037/a0036839 24842069

[B169] Shenhav A. Greene J. D. (2010). Moral judgments recruit domain-general valuation mechanisms to integrate representations of probability and magnitude. *Neuron* 67 667–677. 10.1016/j.neuron.2010.07.020 20797542

[B170] Sherrington C. S. (1906). *The Integrative Action of the Nervous System.* New York, NY: Charles Scribner’s Sons. 10.1037/13798-001

[B171] Showers M. J. C. Lauer E. W. (1961). Somatovisceral motor patterns in the insula. *J. Comp. Neurol.* 117 107–115. 10.1002/cne.901170109 13912292

[B172] Silk J. S. Redcay E. Fox N. A. (2014). Contributions of social and affective neuroscience to our understanding of typical and atypical development. *Dev. Cogn. Neurosci.* 8 1–6. 10.1016/j.dcn.2014.02.002 24613509 PMC6987855

[B173] Singer T. Critchley H. D. Preuschoff K. (2009). A common role of insula in feelings, empathy and uncertainty. *Trends Cogn. Sci.* 13 334–340. 10.1016/j.tics.2009.05.001 19643659

[B174] Singer T. Seymour B. O’Doherty J. Kaube H. Dolan R. J. Frith C. D. (2004). Empathy for pain involves the affective but not sensory components of pain. *Science* 303 1157–1162. 10.1126/science.1093535 14976305

[B175] Singer T. Seymour B. O’Doherty J. P. Stephan K. E. Dolan R. J. Frith C. D. (2006). Empathic neural responses are modulated by the perceived fairness of others. *Nature* 439 466–469. 10.1038/nature04271 16421576 PMC2636868

[B176] Sommer M. Rothmayr C. Döhnel K. Meinhardt J. Schwerdtner J. Sodian B. (2010). How should I decide? The neural correlates of everyday moral reasoning. *Neuropsychologia* 48 2018–2026. 10.1016/j.neuropsychologia.2010.03.023 20362598

[B177] Spreng R. N. Mar R. A. (2012). I remember you: a role for memory in social cognition and the functional neuroanatomy of their interaction. *Brain Res.* 1428 43–50. 10.1016/j.brainres.2010.12.024 21172325 PMC3085056

[B198] Steffel M. Williams E. F. (2018). Delegating decisions: recruiting others to make choices we might regret. *J. Cons. Res.* 44, 1015–1032. 10.1093/jcr/ucx080

[B178] Summerfield J. J. Hassabis D. Maguire E. A. (2009). Cortical midline involvement in autobiographical memory. *NeuroImage* 44 1188–1200. 10.1016/j.neuroimage.2008.09.033 18973817 PMC2625448

[B179] Suzuki M. Kawagoe T. Nishiguchi S. Abe N. Otsuka Y. Nakai R. (2018). Neural correlates of working memory maintenance in advanced aging: evidence from fMRI. *Front. Aging Neurosci.* 10:358. 10.3389/fnagi.2018.00358 30459595 PMC6232505

[B180] Tamber-Rosenau B. J. Asplund C. L. Marois R. (2018). Functional dissociation of the inferior frontal junction from the dorsal attention network in top-down attentional control. *J. Neurophysiol.* 120 2498–2512. 10.1152/jn.00506.2018 30156458 PMC6295539

[B181] Tjew-A-Sin M. Tops M. Heslenfeld D. J. Koole S. L. (2016). Effects of simulated interpersonal touch and trait intrinsic motivation on the error-related negativity. *Neurosci. Lett.* 617 134–138. 10.1016/j.neulet.2016.01.044 26876476

[B182] Tops M. Boksem M. A. S. (2011). A potential role of the inferior frontal gyrus and anterior insula in cognitive control, brain rhythms, and event-related potentials. *Front. Psychol.* 2:33. 10.3389/fpsyg.2011.00330 22084637 PMC3212750

[B183] Van Buuren M. Gladwin T. E. Zandbelt B. B. Kahn R. S. Vink M. (2010). Reduced functional coupling in the default-mode network during self-referential processing. *Hum. Brain Mapp.* 31 1117–1127. 10.1002/hbm.20920 20108218 PMC6870730

[B184] Van Strien N. M. Cappaert N. L. M. Witter M. P. (2009). The anatomy of memory: an interactive overview of the parahippocampal- hippocampal network. *Nat. Rev. Neurosci.* 10 272–282. 10.1038/nrn2614 19300446

[B185] Vernon R. J. W. Sutherland C. A. M. Young A. W. Hartley T. (2014). Modeling first impressions from highly variable facial images. *Proc. Natl. Acad. Sci. U.S.A.* 111 E3353–E3361. 10.1073/pnas.1409860111 25071197 PMC4136614

[B186] Wade-Bohleber L. M. Boeker H. Ernst J. Grimm S. Brügger N. Berwian I. M. (2019). Thinking about the past to shape the present: neural activation during the recall of relationship episodes. *Behav. Brain Res.* 359 783–791. 10.1016/j.bbr.2018.08.001 30077577

[B187] Wagner D. D. (2012). The representation of self and person knowledge in the medial prefrontal cortex. *Wiley Interdiscip. Rev.* 3 451–470. 10.1002/wcs.1183 22712038 PMC3375705

[B188] Wagner D. D. Haxby J. V. Heatherton T. F. (2012). The representation of self and person knowledge in the medial prefrontal cortex. *Wiley Interdiscip. Rev.* 3 451–470. 10.1002/wcs.1183 22712038 PMC3375705

[B189] Wheeler M. A. (2000). “Episodic memory and autonoetic awareness,” in *The Oxford Handbook of Memory*, eds Tulving E. Craik F. I. M. (Oxford: Oxford University Press), 597–608.

[B190] Wicker B. Keysers C. Plailly J. Royet J.-P. Gallese V. Rizzolatti G. (2003). Both of Us Disgusted in My InsulaThe Common neural basis of seeing and feeling disgust. *Neuron* 40 655–664. 10.1016/S0896-6273(03)00679-2 14642287

[B191] Wilbers L. Deuker L. Fell J. Axmacher N. (2012). Are autobiographical memories inherently social? Evidence from an fMRI study. *PLoS One* 7:e0045089. 10.1371/journal.pone.0045089 23028774 PMC3448611

[B192] Winnicott D. W. (1986). “The theory of the parent-infant relationship,” in *Essential Papers on Object Relations*, ed. Buckley P. (New York, NY: New York University Press), 233–253. 10.1111/famp.12023

[B193] Wittmann M. (2015). Modulations of the experience of self and time. *Conscious. Cogn.* 38 172–181. 10.1016/j.concog.2015.06.008 26121958

[B194] Xu M. Li Z. Diao L. Fan L. Zhang L. Yuan S. (2017). Social exclusion impairs distractor suppression but not target enhancement in selective attention. *Int. J. Psychophysiol.* 121 72–79. 10.1016/j.ijpsycho.2017.06.003 28601652

[B195] Young L. Koenigs M. (2007). Investigating emotion in moral cognition: a review of evidence from functional neuroimaging and neuropsychology. *Br. Med. Bull.* 84 69–79. 10.1093/bmb/ldm031 18029385

[B196] Zaytseva Y. Gutyrchik E. Bao Y. Pöppel E. Han S. Northoff G. (2014). Self processing in the brain: a paradigmatic fMRI case study with a professional singer. *Brain Cogn.* 87 104–108. 10.1016/j.bandc.2014.03.012 24732954

[B197] Zhang M. Chen X. Deng H. Lu Z. (2014). Identifying the interaction of maternal sensitivity and two serotonin-related gene polymorphisms on infant self-regulation. *Infant Behav. Dev.* 37 606–614. 10.1016/j.infbeh.2014.06.009 25199967

